# A critical review of anisakidosis cases occurring globally

**DOI:** 10.1007/s00436-023-07881-9

**Published:** 2023-05-26

**Authors:** Shokoofeh Shamsi, Diane P. Barton

**Affiliations:** School of Agricultural, Environmental and Veterinary Sciences, Gulbali Institute, Charles Sturt University, Wagga Wagga, Australia

**Keywords:** Misdiagnosis, Seafood safety, Nematoda, Foodborne parasites, Anisakiasis, Anisakiosis, Anisakidosis

## Abstract

A review was conducted to identify the most common causative agents of anisakidosis, the methods used for identification of the causative agents, and to summarize the sources of infection, and patients’ demographics. A total of 762 cases (409 articles, inclusive of all languages) were found between 1965 and 2022. The age range was 7 months to 85 years old. Out of the 34 countries, Japan, Spain, and South Korea stood out with the highest number of published human cases of anisakidosis, respectively. This raises the question: Why are there few to no reports of anisakidosis cases in other countries, such as Indonesia and Vietnam, where seafood consumption is notably high? Other than the gastrointestinal tract, parasites were frequently found in internal organs such as liver, spleen, pancreas, lung, hiatal and epigastric hernia, and tonsils. There are also reports of the worm being excreted through the nose, rectum, and mouth. Symptoms included sore throat, tumor, bleeding, gastric/epigastric/abdominal/substernal/lower back/testicular pain, nausea, anorexia, vomiting, diarrhea, constipation, intestinal obstruction, intussusception, blood in feces, hematochezia, anemia, and respiratory arrest. These appeared either immediately or up to 2 months after consuming raw/undercooked seafood and lasting up to 10 years. Anisakidosis commonly mimicked symptoms of cancer, pancreatitis, type I/II Kounis syndrome, intussusception, Crohn’s disease, ovarian cysts, intestinal endometriosis, epigastralgia, gastritis, gastroesophageal reflux disease, hernia, intestinal obstruction, peritonitis, and appendicitis. In these cases, it was only after surgery that it was found these symptoms/conditions were caused by anisakids. A range of not only mainly marine but also freshwater fish/shellfish were reported as source of infection. There were several reports of infection with >1 nematode (up to >200), more than one species of anisakids in the same patient, and the presence of L4/adult nematodes. The severity of symptoms did not relate to the number of parasites. The number of anisakidosis cases is grossly underestimated globally. Using erroneous taxonomic terms, assumptions, and identifying the parasite as *Anisakis* (based solely on the Y-shaped lateral cord in crossed section of the parasite) are still common. The Y-shaped lateral cord is not unique to *Anisakis* spp. Acquiring a history of ingesting raw/undercooked fish/seafood can be a clue to the diagnosis of the condition. This review emphasizes the following key points: insufficient awareness of fish parasites among medical professionals, seafood handlers, and policy makers; limited availability of effective diagnostic methodologies; and inadequate clinical information for optimizing the management of anisakidosis in numerous regions worldwide.

**Supplementary Information:**

The online version contains supplementary material available at 10.1007/s00436-023-07881-9.

## Introduction

From the specific identification of the causative agents to the nomenclature of the disease, there is much confusion around the disease caused by anisakid nematodes. In 1988, a standardized nomenclature expert group recommended three different terms: (1) anisakidosis caused by any members of the family Anisakidae, (2) anisakiosis caused by members of the genus *Anisakis*, and (3) pseudoterranovosis caused by members of the genus *Pseudoterranova* (Kassai et al. 1988). However, in the literature, anisakiosis, anisakidosis, and anisakiasis have been used interchangeably and not much attention has been paid to the accurate identification and latest taxonomical changes of these important parasites. In this article, the term anisakidosis is used following the recommendation by the expert group. When referring to the disease caused by *Anisakis* spp., the term anisakiasis was used, due to its global acceptance.

Over one hundred species belong to the family Anisakidae, and the genus *Anisakis* alone comprises of at least 9 species (Shamsi 2021).

Anisakidosis is usually considered a rare condition by medical practitioners (Khan and Williams 2016). In patients with a history of consuming raw or undercooked seafood, the infection is often not included in the differential diagnosis, leading to misdiagnoses (Roser and Stensvold 2013; Shimamura et al. 2016; Shamsi and Sheorey 2018; Seal et al. 2020). Even if the infection is diagnosed, the causative agent is usually referred to as a member of the family Anisakidae, most commonly *A. simplex* or *A. pegreffii*, without providing evidence for the identification. While some may argue that the specific identification of the parasite may not be clinically important, the true incidence of these infections and clinical syndromes associated with various species will never be documented properly if not identified correctly. Additionally, risk assessments of food products and food safety policies rely on the correct identification of pathogens (Huss 2007). This lack of knowledge is likely to cause major shortfalls in the efficacy of risk assessments, policy development, and international trade of safe seafood (Shamsi 2019). This in turn has the potential to impact patient care and public health negatively.

The aims of this review were to determine the most common causative agents of anisakidosis and the methods used for identification of the causative agents, and to summarize the sources of infection and patients’ demographics.

## Materials and methods

The review was conducted to provide demographic information (including age, gender, and country) of infected people with anisakid nematodes and to answer these questions: What are the parasite species reported as the causative agent? What was the possible source of infection? What method was used to identify the parasite and/or diagnose the disease? What symptoms were presented? The review was based on the publicly available information. A review of peer-reviewed literature for topics on Anisakiasis, OR Anisakidosis OR Anisakiosis, was conducted through a search of the Google Scholar, Web of Science, PubMed, and Scopus databases. The bibliographies of the articles found through the search were checked for any other articles relevant to the topic. All languages were included. Following this, some gray literature, such as conference abstracts and proceedings, were also included after ensuring there was no associated publication, acknowledging the lack of peer review for this type of literature. The literature was last searched on 1st February 2023. Although *Hysterothylacium* spp. are now included in the Raphidascarididae family (Deardorff and Overstreet 1981; Nadler et al. 2005), they were included in this study because they used to be considered members of the family Anisakidae in the past. All languages were included. Non-English articles were translated using Google Translate app and, if needed, help was sought from a native speaker. Cases dealing with parasite misidentification and duplicates were removed manually by reviewers, and records were screened by going through the title, abstract, and full-text screening. When there was a doubt about an article decision, we were inclusive rather than exclusive, until a decision was made after discussion and consensus (Tawfik et al. 2019). In articles with two or more cases, each case was counted separately. If the same patient, more than once, in different years, was diagnosed with the disease, each time was counted as separate case. Where the same case has been published more than once by different authors (Richman and Lewicki 1973; Pinkus et al. 1975; Valdiserri 1981; Appleby et al. 1982), the first publication was considered for the publication year. Allergic anisakiasis is not in the scope of this review.

Data including authors, year of publication, country, age, and gender of the patient, diagnostic techniques for the disease, method of parasite identification, parasite ID, developmental stage of the parasite, number of parasites found in the patient, infected organs, symptoms, treatment, the time between consuming seafood and emergence of the symptoms, duration of the symptoms, and possible source of infection were recorded in an MS Excel sheet.

## Results

### Case reports

**Fig. 1:**
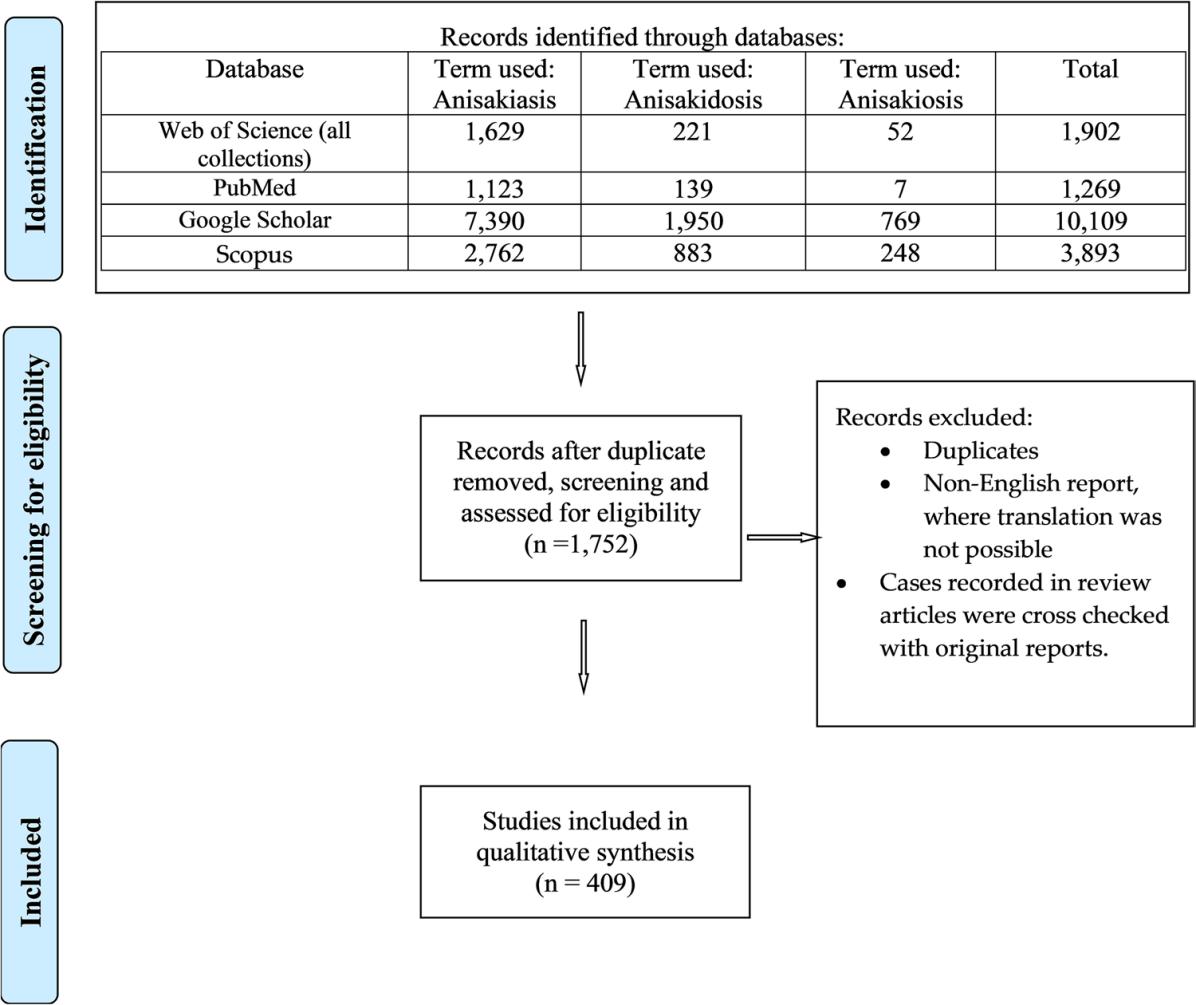
The literature search through this study

**Fig. 2:**
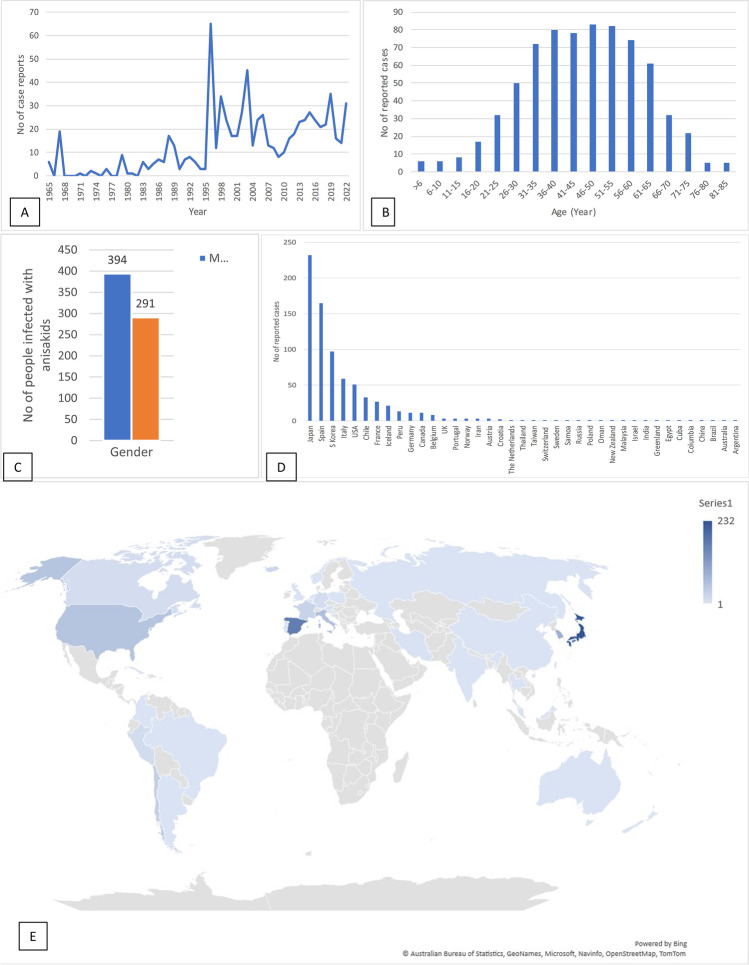
Diagrams summarizing the demographic data found in the present study. **A** Number of reports of anisakidosis cases in different years. **B** Number of reported of anisakidosis cases in different age ranges. **C** Percentage of females and males found to be infected with anisakid nematodes during the study period. **D** and **E** Number and distribution of anisakidosis cases in different countries. The country in which the disease was diagnosed was considered the place of the occurrence of anisakidosis, including a case of a Japanese man visiting the USA and travelers who recently returned from Brazil and Portugal

A total of 409 articles were included in the present study (Fig. 1). Herein, the term anisakidosis will be used to refer to the disease caused by anisakid larvae, inclusive of *Hysterothylacium* spp. These 409 articles reported 762 cases dated between 1965 and the end of 2022 (Fig. 2A).

### Age and gender of infected people

Where recorded in the reported cases (*n* = 713), it was found that the minimum and maximum age of infected people was 7 months old and 85 years old (Fig. 2B). A total of 291 (42%) of the infected people were reported as females and 394 (58%) as males (Fig. 2C).

### Global reports

Anisakidosis cases were reported in 34 countries (Fig. 2D and E), with Japan, Spain, South Korea, Italy, and the USA, being the top five countries with the highest number of published human cases, respectively.

**Fig. 3:**
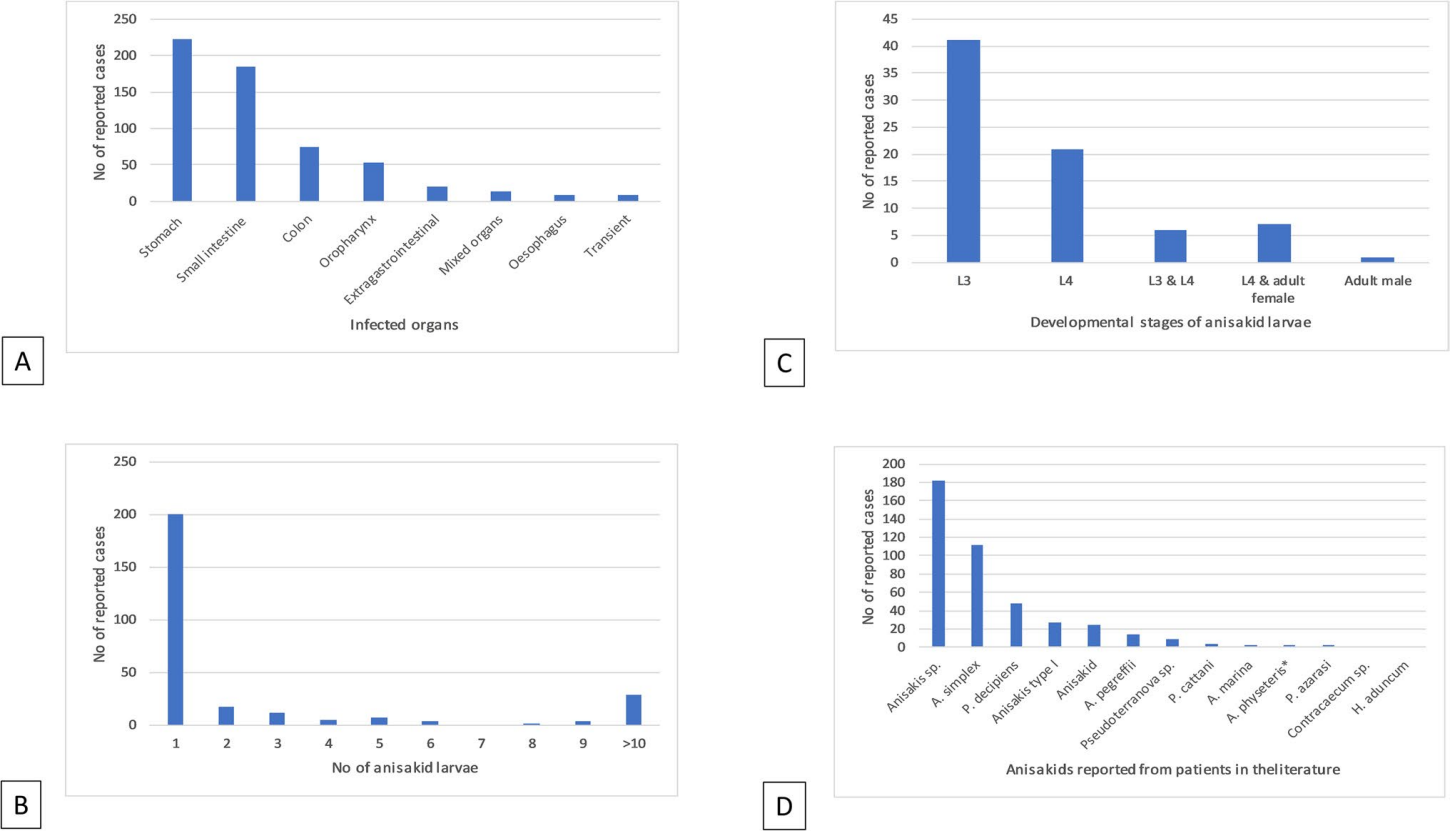
Diagrams summarizing the data found in the present study. In panel **A**, transient includes reports of the live parasite exiting human body. This included when it was reported in the sputum, feces, diaper, nose, and mouth and one case where the worm was reported to exit from the skin around neck; extra gastrointestinal includes mesentery, liver, spleen, pancreas, lung, lymph nodes, scrotum, uterus, ovary, glands around anus, found in dialysis affluent, found in a nodule on the large omentum and amyand, hiatal and epigastric hernia; one case of polyarthritis caused by anisakid nematode was also placed under gastrointestinal; and oropharynx includes larynx, tonsil, tongue, and throat. Appendicitis cases were considered under small intestine. Mixed organ infection included stomach and colon (*n* = 1), stomach and small intestine (*n* = 8), stomach and colon (*n* = 3), and stomach and esophagus (*n* = 1). Panel **B** shows the number of anisakid larvae found in infected people. Panel **C** shows the frequency of various developmental stages of anisakid larvae found in infected people. In panel **D**, anisakids includes reports as anisakids, Anisakidae, Anisakine type, *Anisakis* like larva, and two cases where authors were not sure between *Anisakis* or *Pseudoterranova* and *Pseudoterranova* or *Contracecum*. *Anisakis* sp. includes reports in which parasite referred as *Anisakis* sp., and *Anisakis*; *A. simplex* includes reports of *A. simplex*, *A. simplex sensu lato*, and *A. simplex sensu stricto*; *Pseudoterranova decipiens* includes *Phocanema decipiens*, *Pseudoterranova decipiens sensu stricto*, and *Pseudoterranova decipiens sensu lato*

The most common infected organs were the stomach and small and large intestines (Fig. 3A). However, parasites were commonly found in other organs, such as the liver, spleen, pancreas, lung, scrotum, uterus, ovary, hernia, mesentery, lymph nodes, and tonsils. They were also reported exiting the body through the nose, rectum, sputum, and mouth. There were some unusual reports of extra gastrointestinal anisakidosis, including a case where anisakid larva was found in continuous ambulatory peritoneal dialysis (CAPD) effluent and also a case in which anisakid larva was extracted from patient’s neck.

### Symptoms and clinical signs

Of 48 asymptomatic cases reported, diagnosis was made accidentally, often during a regular check-up for other conditions, such as rectal or stomach cancer. In symptomatic cases, symptoms mentioned in literature included numerous specific and non-specific signs and symptoms (Table 1). Gastrointestinal manifestations were the commonest.

**Table 1:** The variety of presentations mentioned in the various references

GI manifestations	Dysphagia, edema of epiglottis, foreign-body sensation, sensation of sticking (in mouth), oral pain, burning mouth, lip angioedema, ulcerative desquamative gingivitis, erosive inflammatory lesions of the labial mucosa gastric bleeding, gastric/epigastric pain, burning, nausea, anorexia, incarcerated inguinal/epigastric hernia dyspepsia regurgitation, vomiting, hematemesis, decreased/loss of appetite, weight loss, abdominal cramp and discomfort, abdominal pain diarrhea, constipation, intestinal obstruction, enteritis, feeling full, flatulence, intussusception positive fecal occult blood test , melena, blood in feces, rectal bleeding large amount of blood in stool
Respiratory manifestations	Dyspnea sensation of asphyxia, sore throat respiratory symptoms (including coughing, wheezing, shortness of breath), respiratory arrest, acute retrosternal, substernal pain, tingling in the pharynx/throat, throat/pharyngeal pain
CNS manifestations	Headache, a loss of consciousness
Constitutional signs/symptoms	Fever, sweating, hypovolemic shock, anaphylaxis
Signs/symptoms suggestive of allergic reactions	Erythematous hives, erythema and edema, urticaria, itchiness, diffuse urticarial papules wheals
Other/non-specific signs/symptoms	Tumor-like lesion, arthritis of knees, elbows and ankles, rapidly growing left inguinal mass, lower back pain, groin pain, testicular pain, periumbilical pain edema of arytenoid, asthenia, intense cold, low blood pressure, hematochezia, anemia, facial rash, rash on arm/chest/back, pruritic micro-vesicular lesions, interphalangeal and palmar fissures, nephrotic syndrome

### Characteristics of the causative agents

Of cases in which the number of parasites was reported, most cases were due to one parasite (*n* = 200). In 27 cases in which more than 10 anisakid larvae was reported (Fig. 3B), there were cases with 50, 56, 140, and over 200 larvae in one patient. Most articles did not mention the developmental stage of the parasite. Based on the reported cases, the third stage of the larval development was the most common among anisakidosis cases (Fig. 3C), followed by the fourth stage of the development and adult stage, respectively, with some cases in which more than one developmental stage was found in one patient.

The identification of the parasite was mentioned in 413 cases, with the rest only reporting cases of anisakiasis/anisakidosis/anisakiosis without naming the causative agent (Fig. 3D). Anisakid species to cause human infections in the 413 cases were reported as Anisakidae, anisakid, anisakine type, *Anisakis* sp., *Anisakis*, *Anisakis* type I, *A. simplex*, *A. simplex s. s.*, *A. simplex s. l.*, *A. pegreffii*, *A. marina*, *A. physeteris*, *Contracaecum* sp., *Pseudoterranova*, *Pseudoterranova azarasi*, *Pseudoterranova cattani*, *Pseudoterranova decipiens*, *P. decipiens s. l.*, *P. decipiens s. s.*, *Phocanema*, *Phocanema decipiens*, and *Hysterothylacium aduncum*.

### Diagnosis

There were 159 reports of misdiagnosis that were eventually diagnosed as anisakidosis. Some examples include where patients were initially diagnosed with cancer (including liver metastasis from rectal cancer, metachronous liver metastasis of testicular cancer, gastric cancer, duodenal carcinoma, colon cancer, colorectal cancer, pancreas cancer, and complicated tumor), acute biliary pancreatitis, autoimmune pancreatitis, type I and II Kounis syndrome, colonic intussusception, Crohn’s disease, enlarged bilateral ovarian cysts, intestinal endometriosis, epigastralgia, gastritis, gastroesophageal reflux disease, hernia, intestinal obstruction, ileum to ileum intussusception caused by Meckel’s diverticulum, peritonitis, acute appendicitis, acute arthritis of knees, elbows and ankles, and non-anisakid parasitic disease.

### Possible source of infection

The source of infections, based on the patients’ reports, was a wide range of seafood. These included marinated anchovies (known as “boquerones en vinagre,” or raw anchovies (*Engraulis encrasicolus*) pickled in vinegar, a typical Mediterranean food), croaker fish, fish swimbladder, raw mackerel, abalone, penis fish, assorted sashimi (horse mackerel, mackerel, sea bream, flounder, octopus, and shellfish), baked cod, catfish, ceviche, cod liver, conveyor belt sushi (tuna, yellow tail, and whelk), raw sea bream, eel sashimi, empurau fish (*Tor tambroides*), fish eggs, flatfish, yellowtail, flounder sashimi, fresh cured Alaskan salmon, poke style raw tuna from Alaska, grilled scabbard fish, hake, herring fish, home-cured salmon gravlax, marinade bluefish, marinated mackerel, marinated pilchards, raw Atlantic salmon (*Salmo salar*), raw bonito, raw clams, raw cuttlefish, raw oyster, raw Pacific saury, raw salted fish or squid (mackerel, squid, sardine, saury, scallop, sea bream, bonito, flounder, or a variety of fish prepared as sushi), shrimp, raw yellowtail, redfish/sushi, rockfish (*Sebastes* sp.), fresh slices of raw jacopever, shabu-shabu (mackerel fish), trout, sliced raw fish (*Sebastes schlegelii*), ascidians, and a piece of flatfish gut, prior to the disease occurrence. The source of these fish was not only mainly from marine waters but also occasionally from freshwater and local rivers. Based on the history provided by patients, the onset of the symptoms was highly variable, starting from immediately to 2 months after consumption of the abovementioned seafood. In one case, the duration of symptoms was up to 10 years of intermittent pain.

## Discussion

Studies published in languages other than English are often neglected in most recently published reviews and systematic reviews on anisakids. Due to inclusion of the old literature inclusive of all languages (Supplementary Table), we were able to find some of the overlooked/forgotten knowledge about anisakid nematodes, as discussed below.

Two publications reporting *Anisakis* in the oral cavity were found to be misdiagnoses (Song et al. 2006; Choi et al. 2017). Images provided in these two articles suggest that authors confused infection with the sperm bag of squid or tapeworm larvae with anisakidosis. These two articles (Song et al. 2006; Choi et al. 2017) were excluded from this review.

### Case reports and articles

Despite a clear decline in the number of reported cases during 2020 (due to the impact of COVID-19 pandemic), and considerable variations in the pattern of occurrence by year, the number of publications is clearly higher since the beginning of the millennium. Although we included publications since 1965, there are reports of human cases dating back to the nineteenth century. For example, in 1867, a nematode had been vomited by a child from a fishing community on the West Coast of Greenland and identified as *Ascaris maritima* (Leuckart 1876). *Ascaris maritima* is an invalid species and now is considered an anisakid nematode (Martin 1921). In another example, in 1950, nematodes referred as anisakines were found in the feces of people from Alaska (Hitchcock 1950; Jackson 1975). In Japan, an intestinal disease causing severe allergic tissue reaction with extensive eosinophilic infiltration, and sometimes with cross sections of a nematode-like worm in histological samples, was known since 1950 (Ishikura et al. 1993). In 1960, in the Netherlands, van Thiel et al. (1960) found the larva of a nematode in the intestinal wall of a patient with history of eating raw herring, who was presented with an acute abdominal syndrome. However, it was in 1962 that the disease was named anisakiasis (Van Thiel 1962). The total number of anisakidosis cases worldwide was predicted to be around 76,000 by 2017 (Bao et al. 2019); however, considering that Japan, alone, has an average of 19,737 anisakiasis/anisakiosis cases per year (Sugiyama et al. 2022), in addition to the points mentioned above, this number is most likely a significantly underestimation.

### Age and gender of infected people

Our review suggests that, globally, the parasite is more common among people aged 31-60 years, similar to the age groups reported in country-focused studies. Cha and Mee Sun (2012), based on case reports published from 2000 to 2010, showed that the incidence age changed from the 30s and 40s to the 50s. This could be due to the difficulties in diagnosis in other age ranges (e.g., children) and more exposure to parasites in adults aged between 31 and 60 years. Studies on the actual prevalence rate among children are lacking. In regard to the gender, Yera et al. (2018) conducted a national retrospective survey of anisakidosis in France (2010–2014) and showed a female predominance of infections. They found only 37 cases in 4 years and only 7 of them were diagnosed by finding larvae; therefore, these results should be approached with cautiously. In another study in Japan, Oshima (1972) reported the disease to be more abundant in males than in females (2-2.5:1) and assigned the high rate in male to the frequent eating of raw seafood with alcoholic beverages. In our review, males were more commonly infected than women globally.

### Global reports

As previously shown by other authors (Guardone et al. 2018a; Rahmati et al. 2021; Suzuki et al. 2021a), our review also shows that Japan accounts for most anisakidosis cases. This raises the question—why have there been no/few reports of anisakidosis cases in other south-eastern Asian countries (Wiwanitkit and Wiwanitkit 2016), such as the Philippines, Myanmar, Cambodia, Vietnam, Indonesia, Malaysia, and Brunei, or in the Pacific region, where seafood consumption is high and it is the main protein source in many of these countries? Anisakid larvae are commonly found in the edible fishes in south-eastern Asian countries (Arthur and Lumanlan-Mayo 1997; Palm et al. 2017) and also in the Pacific region (Shamsi et al. 2015; Shamsi et al. 2018). Similarly, in other parts of the world, such as in Scandinavian countries, fish are found to be heavily infected (Zuo et al. 2017) but the number of human cases is low (Eskesen et al. 2001). This observation suggests that there could be other unknown factors involved, rather than merely a tradition of frequent consumption of raw fish in these regions, as previously pointed out by many authors. It has been suggested anisakidosis is not frequent in China, because the Chinese usually eat the fish raw at the end of the meal, whereas the Japanese eat raw fish at the start of the meal when the stomach is empty (Chao 1985). Another important contributing factor for more case reports in Japan might be due to cancer control programs. Cancer screening for gastric, colorectal, lung, breast, and cervical cancers are regularly conducted in Japan (Hamashima 2018; Yashima et al. 2022), which may have led to increased awareness of the parasite among the health professionals, and hence more cases being reported. Additionally, our review suggests that where there is awareness and knowledge among the medical and health professionals, as is in Japan, Spain, and South Korea, an accurate diagnosis is likely to be made. Consumption of raw or undercooked seafood is steadily increasing globally (Huang and Bussieras 1988; Golden et al. 2022). Japan, Spain, and South Korea are not unique in terms of increased seafood consumption as increased human movements and changing food habits are happening globally.

Anisakidosis is increasingly considered a relatively common public health issue in many countries. Previously it was estimated that more than 2500 cases occur annually in Japan, 20 cases per country per year (AFSSA 2011) or 0.32/100,000 (Una-Gorospe et al. 2018) in Europe, and 10 cases per year in the USA (AFSSA 2011); however, a recent study based on insurance claims showed that an average of 19,737 anisakiasis cases per year occur in Japan (Sugiyama et al. 2022). Therefore, the exact frequency of anisakidosis is unknown. Some authors (Herrador et al. (2019); Morozinska-Gogol (2019)) considered Spain the second country after Japan with the highest number of cases of anisakidosis. In other countries, such as Morocco (Abattouy et al. 2012), however, although no cases of human anisakidosis have been reported, positive serological tests indicate previous exposure to the live larva. Because the reporting of cases is not mandatory, further suggests that the disease is underestimated and the numbers reported in different countries might be different from the actual occurrence.

### Infected organs, symptoms, and clinical signs

As previously reported by several authors (Ishikura et al. 1993), we also found that gastric anisakidosis is the most common form of anisakidosis followed by intestinal anisakidosis. Our review shows that anisakids can be found in almost any organ in the human body. Since the site of the parasite penetration determines the clinical signs and symptoms, a wide range of symptoms and clinical signs were observed (see the “Results” section and Table 1). The severity of pain caused by anisakids has been described as 10 out of 10 by some patients. There are reports that despite the effect of strong pain killers such as morphine, the patient still felt intense abdominal pain (Daschner et al. 1997). The highly variable onset of the symptoms found in the literature along with the duration of symptoms to up to 10 years (Yokogawa and Yoshimura 1965; Pinkus et al. 1975; Maggi et al. 2000) adds to the challenges of accurate and timely diagnosis. It was always thought anisakid larvae may live in humans for up to 8 weeks (Jackson 1975; Eskesen et al. 2001). It is not known if long duration of the symptoms is a matter of chronic anisakidosis or recurrent anisakidosis. Also, there might not be a correlation between the number of parasite and the severity of symptoms as a patient with 11 larvae was asymptomatic (Hokama et al. 2005). Generally, larvae belonging to the genus *Pseudoterranova* are noninvasive, and cause transient or oropharyngeal anisakidosis (Fukui et al. 2020; Cha et al. 2022) but can also be invasive and penetrate host tissues (Pinel et al. 1996; Yu et al. 2001). *Anisakis* spp. are most often found in the mucosa or submucosa of the stomach and intestine (Rosales et al. 1999; Dominguez Ortega et al. 2000) (Vercammen et al. 1997; Maggi et al. 2000; Sohn et al. 2015) and have migrated to other tissues, such as the omentum (Cancrini et al. 1997), pancreas (Brandt 2014), liver (Nogami et al. 2016), and lung (Kobayashi et al. 1985).

We could not find any death due to infection with anisakid nematodes in the published literature; however, there are anecdotal deaths due to misdiagnosis and/or anaphylaxis after undergoing anesthesia for acute abdomen.

### Causative agents and diagnostic issues

The third stage larval development of anisakids has always been considered the infective stage of the parasite. It has been suggested that when an *Anisakis simplex* larva is cut into two pieces, the anterior part is still able to penetrate the wall of the digestive tract (Asami and Inoshita 1967). Most authors state that no further development of anisakid larvae occurs in human hosts (Jackson 1975). However, during the present review, several reports of fourth stage of larval development and also adult stage of anisakids (including *Anisakis* and *Pseudoterranova*) were reported in humans (Kliks 1983; Kowalewska-Grochowska et al. 1989; Pinel et al. 1996; Mercado et al. 2001; Suzuki et al. 2021b). Development of *A. pegreffii* and *A. berlandi* larvae from L3 to L4 has also been reported in birds (Johnston and Mawson 1942; Shamsi et al. 2017) which suggests broader host specificity of the parasite than previously thought.

In the literature, anisakidosis is known to be caused mostly by the larval stage of anisakid nematodes belonging to three genera, *Anisakis*, *Contracaecum*, and *Pseudoterranova*. Of over 9 species belonging to the genus *Anisakis*, three species *A. pegreffii*, *A. simplex* s. s*.*, and *A. physeteris* have been reported from humans. The latter has been identified to species based on the occurrence/abundance of the parasite in the environment where human infection occurred (Cabrera and Suárez-Ognio 2002), whereas the first two species were identified based on the molecular sequencing of the larvae collected from humans (Umehara et al. 2007). Both *A. simplex* s. s. and *A. pegreffii* have demonstrated their ability to cause anisakiasis in humans, but based on the effect of the holding temperature on penetration of their larvae into fish muscle tissue and also their ability to penetrate into agar, it has been suggested that *A. simplex s. s.* penetrates the muscle of the fish at a higher rate than *A. pegreffii* and therefore, poses higher risk to human health (Suzuki et al. 2010).

It has been argued that the absence of other *Anisakis* species in human cases (Mattiucci et al. 2018) might be due to their preferential infection sites in the body cavity of the fish hosts (being encysted on visceral organs). While we agree, however, our review also suggests that this might be simply due to no attempt by health professionals to specifically identify larvae in human cases on morphological criteria alone. Similarly, to the best of the authors’ knowledge, *C. osculatum*’s specific identification of larvae from humans is based on morphology rather than molecular confirmation. Molecular tools such as sequence of the rDNA region or other molecular-based diagnostic techniques are necessary to accurately identify parasite larvae (Nascetti et al. 1983; Paggi et al. 1991; Shamsi et al., 2011). We believe, more anisakid species could be identified as causing the disease if adequate steps to identify the pathogens were taken. Studies suggest *Anisakis* spp. are the most common cause of anisakidosis in humans, followed by *Pseudoterranova* spp. (including *P. decipiens*, *P. cattani*, and *P. azarasi* (Skirnisson 2006; Arizono et al. 2011; Weitzel et al. 2015), *Contracaecum* spp. (Shamsi and Butcher 2011), and *Hysterothylacium* spp. (Gonzalez-Amores et al. 2015). The present review shows that in most human case reports, parasite identification has not been verified by molecular techniques. For example, our results show that of reported cases included in the study, only 7% mentioned using molecular tools for identification of the parasite. Of those that provided details of the identification, many are questionable due to identification of species based on the morphology alone.

We also found that it is common practice to identify anisakid larvae in histopathological examinations as *Anisakis* larva, based on a Y-shaped or butterfly-like lateral cord in the parasite cross section (Pampiglione et al. 2002; Testini et al. 2003); this is concerning because several other members of the Anisakid nematodes, including *Pseudoterranova*, also have a Y-shaped lateral cord (in cross-sectional examination) (Gibson 1983), suggesting the number of cases due to *Pseudoterranova* spp. larvae and other anisakids may have been significantly underestimated in the literature. Distinctive Y-shaped lateral chords, no lateral alae extending from cuticle, 60 to 90 muscle cells per quadrant and 60 to 80 cells in the intestine, are the characteristics to be used to identify *Anisakis* larvae in histological sections. *Pseudoterranova* spp. larvae have more than 100 intestinal cells, and an intestinal cecum (Oshima 1972). The absence of mention regarding the presence of a Y-shaped lateral cord in any of the reported cases might be attributed to the need for patience and expertise in accurately counting intestinal cells. Although some may believe specific identification of the parasite may not be important for management of the case, it is difficult to be sure unless the confusion around nomenclature and diagnoses are addressed. There is no clinical study to address if the different symptoms and pathologies are due to different species of parasites. Additionally, risk assessments of food products and food safety policies rely on the correct identification of pathogens.

The case of human infection with *Hysterothylacium* larva is also important (Gonzalez-Amores et al. 2015). There have been debates among researchers about the pathogenicity and zoonotic potential of *Hysterothylacium* nematodes (Roca-Geronès et al. 2018). Accurate identification of the parasite in human cases therefore is essential, to shape the future of dealing with the parasite in policies for fisheries, food safety guidelines, and other disciplines (Shamsi 2014, 2019). Based on experimental infection, some authors suggested the *Hysterothylacium aduncum* larvae do not produce eosinophilic granulomas observed in anisakidosis patients because they could not perform an evolution in homeothermal animals (Vermeil et al. 1975). However, in another experiment, within a few hours after being administered to the rhesus monkey (*Macaca mulatta*), *Hysterothylacium* larvae penetrated the stomach wall, causing hemorrhage and attracted eosinophils (Overstreet and Meyer 1981). We found two reports of human infection with *Hysterothylacium*. It is important to remember that third and fourth larval and adult stages of *Hysterothylacium* spp. all occur in fish. Yagi et al. (1996) reported human infection with adult female *Hysterothylacium* in a 55-year-old man. They used morphological and scanning electron microscopical examination and identified the worm as *Hysterothylacium aduncum*. The information provided in the paper is sufficient to accept the identification of the adult parasite as belonging to the genus *Hysterothylacium*, but it is not sufficient to accept the species identification as proposed by the authors, as *H. aduncum*. In another paper, Gonzalez-Amores et al. (2015) used light and scanning electron microscopy to identify a parasite as a *Hysterothylacium* larva. In this case, even the genus identification is dubious due to the lack of details in the information provided. There are still many unknowns about the epidemiology of the disease and underestimating the value of accurate identification of human cases. Worse of all, any misdiagnosis can hinder/misguide our progress toward better understanding various aspects of the disease and effective prevention and treatment. Without accurate specific identification of larvae causing human anisakidosis, important questions remain unanswered; e.g., why is human anisakidosis mostly mild in some people but severe in others? Why do some larvae undertake abnormal tissue migration in humans but others do not? and do larvae of certain species produce particular clinical signs?

Accurately identifying the parasite in anisakidosis cases is also crucial to explain the different patterns of the disease observed in anisakidosis cases. For example, most Japanese patients infected with *Pseudoterranova* spp. had acute or subacute abdominal pain (Arizono et al. 2011) and Korean patients complained of epigastric pain (Yu et al. 2001) and the parasite expelled from the mouth or rectum. Yet no gastric symptoms were reported in other cases and countries (Na et al. 2013; Skirnisson 2022). It is recognized that diagnostic error data are sparse (Murphy 2016). Patients and their relatives have so much to lose when there is a misdiagnosis (Suzuki et al. 2022). In many cases, patients are subjected to invasive treatments, such as laparoscopic sigmoidectomy and partial resection of ileum, whereas several reports (Takano et al. 2013; Morikawa and Hiraoka 2014) have shown that conservative treatments can be effective.

### Disease mechanism

Our knowledge on the disease mechanism, clinical features, and immunological aspects caused by anisakids in humans is poor. Some studies suggested *Anisakis* infection might be a risk factor for the development of stomach or colon cancer (Garcia-Perez et al. 2015). A limited relation between gastric cancer mortality and the consumption of salted fish presumably contaminated by *Anisakis simplex* (Petithory et al. 1990) was also found. In an early work on anisakiasis, low molecular weight fraction of extract with “tumor-promoter-like activity” was reported in *Anisakis* (Desowitz 1986). In one case, based on the unusual absence of the symptoms in an AIDS patient, authors suggested that the immunodeficiency had influenced the pathogenesis of the parasitic disease (Spehn et al. 1988). Almost all of these studies are focused on *Anisakis* species with little or no knowledge on other members of anisakid nematodes.

### Source of infection

All reported cases had history of eating raw/undercooked/microwaved seafood, including freshwater and marine fish and shellfish. Anisakid larvae are not host specific in their larval stages and have been frequently reported in the seafood listed in the “Results” section from many regions of the world, with more frequency in fish than shellfish species (Karl 2008; Kijewska et al. 2009; Chen et al. 2010; Angelucci et al. 2011; Bernardi et al. 2011; Choi et al. 2011; Madrid et al. 2012; Shamsi et al. 2016; Guardone et al. 2018b; Hossen and Shamsi 2019; Suthar and Shamsi 2021; Williams et al. 2022). Global warming and climate change are also reported to cause a rise/shift in the population of these parasites in fish/shellfish (Shamsi et al. 2018; Fiorenza et al. 2020b; Shamsi 2021). Another important point to consider is that most reports of sources of infection only stated the common name of the fish which can be confusing for efficient risk mitigation strategies (Sumner et al. 2015). Moreover, the broad scale global issues of fish nomenclature and substitution (Williams et al. 2020a, b) may have serious and complicated consequences for consumers. Anisakids and other transmissible parasites in substituted fish may pose a human health risk. Health professionals should be made aware, in cases of fish-borne human illness such as anisakidosis, and clinical presentation may not match the parasite profile of the fish the consumer is believed to have consumed.

## Conclusions

The number of anisakidosis cases is on the rise. They are reported from more countries across the world. There are also growing evidence on the increasing prevalence and abundance of infectious stages of anisakids in seafood (Zuo et al. 2017; Fiorenza et al. 2020a). However, it is concerning that still in many countries the curriculum in veterinary and medical schools is not adequate for educating health professionals about these parasites and the diseases caused by them (Seal et al. 2020; Bradbury et al. 2022). A significant number of misdiagnoses as well as misidentification of the parasite occur globally. This is mainly because diagnosis has been made on morphological criteria alone, which has its own limitations including dwindling number of parasitology morphologists (Bradbury et al. 2022). There are still many unknowns about anisakidosis and its causative agents. Little is known about the impact of anisakids on pregnant women, children, people with immune system deficiencies, and the true global burden of anisakidosis. We need to increase awareness of fish parasites among medical professionals and anyone handling edible seafood, have better diagnostic methods easily available, and carry out well-designed clinical studies to optimize management of anisakidosis.

## Supplementary information

ESM 1 (DOCX 74 kb)
