# Supplementary material for: A critical review of anisakidosis cases occurring globally
Source: Parasitol Res. 2023 May 26;122(8):1733–45. doi: 10.1007/s00436-023-07881-9 (PMC10213583; doi:10.1007/s00436-023-07881-9)
Supplement: Supplementary file 1 — (DOCX 74 kb) [file 436_2023_7881_MOESM1_ESM.docx]

Supplementary table

| Abe K, Yoshikai T, Baba S, Isoda T, Honda H (2014) PET/CT Findings in Acute Gastric Anisakiasis. Clinical Nuclear Medicine 39(6):E340-E342 |
| --- |
| Acebes Rey JM, Fernandez Orcajo P, Diaz Gonzalez G, Velicia Llames R, Gonzalez Hernandez JM, Citores Gonzalez R (1996) 2 cases of anisakiasis in the Del Rio Hortega Hospital (Valladolid). Revista espanola de enfermedades digestivas : organo oficial de la Sociedad Espanola de Patologia Digestiva 88(1):59-60 |
| Agarwal D, Dutta S, Paranjape C (2022) Intramural Intestinal Nematode Causing Small Bowel Obstruction. Indian Journal of Surgery doi:10.1007/s12262-022-03478-1 |
| Ahmed M, Ayoob F, Kesavan M, Gumaste V, Khalil A (2016) Gastrointestinal Anisakidosis - Watch What You Eat. Cureus 8(11) doi:10.7759/cureus.860 |
| Akasaka Y, Kizu M, Aoike A, Kawai K (1979) Endoscopic management of acute gastric anisakiasis. Endoscopy 11(2):158-162 doi:10.1055/s-0028-1098343 |
| Allison C, Baumann M (2019) A case report of adhesional small bowel obstruction caused by extraintestinal anisakiasis. Journal of Emergency Medicine 57(6):E175-E179 doi:10.1016/j.jemermed.2019.08.038 |
| Alonso A, Daschner A, MorenoAncillo A (1997) Anaphylaxis with Anisakis simplex in the gastric mucosa. New England Journal of Medicine 337(5):350-351 doi:10.1056/nejm199707313370518 |
| Alonso A, Moreno-Ancillo A, Daschner A, Lopez-Serrano MC (1999) Dietary assessment in five cases of allergic reactions due to gastroallergic anisakiasis. Allergy 54(5):517-520 doi:10.1034/j.1398-9995.1999.00046.x |
| Alvarez Delgado A (2020) Endoscopic submucosal dissection of colonic anisakiasis COMMENTARY. Revista Espanola De Enfermedades Digestivas 112(10):808-808 |
| Amano M, Fukumoto A, Yamao K, Imagawa H, Hashimoto Y, Iiboshi T, Onogawa S, Hirano N, Hanada K, Yonehara S (2013) Successful treatment of enteric anisakis through endoscopic extraction using a double-balloon enteroscope. Gastroenterol Endosc 55:1643-1649 |
| Amin OM, Eidelman WS, Domke W, Bailey J, Pfeifer G (2000) An unusual case of anisakiasis in California, USA. Comparative Parasitology 67(1):71-75 |
| Amir A, Ngui R, Ismail WHW, Wong KT, Ong JSK, Lim YAL, Lau Y-L, Mahmud R (2016) Case Report: Anisakiasis Causing Acute Dysentery in Malaysia. American Journal of Tropical Medicine and Hygiene 95(2):410-412 doi:10.4269/ajtmh.16-0007 |
| Amo Pelaez M, Munoz Codoceo C, Martinez Montiel P, Sanchez Gomez F, Castellano G, Solis Herruzo JA (2008) Multiple anisakiasis. Revista Espanola De Enfermedades Digestivas 100(9):581-582 |
| Andrisani G, Spada C, Petruzziello L, Costamagna G (2014) An unusual colonic "tumour". Digestive and Liver Disease 46(5):477-478 doi:10.1016/j.dld.2014.01.002 |
| Aparicio Tijeras C, Ezquerra Gadea J, Lopez Larrayoz I, Sanchez Ruiz JC (2004) General pruritus caused by Anisakis. Atencion primaria 34(4):211-2 |
| Appleby D, Kapoor W, Karpf M, Williams S (1982) Anisakiasis - nematode infestation producing small bowel obstruction. Archives of Surgery 117(6):836-836 ; Valdiserri RO (1981) Intestinal anisakiasis - report of a case and recovery of larvae from market fish. American Journal of Clinical Pathology 76(3):329-333 |
| Apt W, Hisamoto T, Llorens P, Alcaino H (1980) Gastric anisakiasis. Revista Medica De Chile 108(9):825-827 |
| Arai T, Yamada H, Edagawa T, Sugiyama H, Nakachi K (2019) Easy detection and fast removal of gastric Anisakis during narrow-band imaging endoscopy with l-menthol administration. Case Reports in Gastroenterology 13(2):305-309 doi:10.1159/000501067 |
| Arenal Vera JJ, Marcos Rodriquez JL, Borrego Pintado MH, Bowakin Dib W, Castro Lorenzo J, Blanco Alvarez JI (1991) Anisakiasis as a cause of acute appendicitis and rheumatologic picture: the first case in medical literature. Revista espanola de enfermedades digestivas : organo oficial de la Sociedad Espanola de Patologia Digestiva 79(5):355-8 |
| Asami K, Watanuki T, Sakai H, Imano H, Okamoto R (1965) 2 cases of stomach granuloma caused by Anisakis-like larval nematodes in Japan. American Journal of Tropical Medicine and Hygiene 14(1):119-& doi:10.4269/ajtmh.1965.14.119 |
| Audicana MT, Decorres LF, Munoz D, Fernandez E, Navarro JA, Delpozo MD (1995) Recurrent anaphylaxis caused by Anisakis simplex parasitizing fish. Journal of Allergy and Clinical Immunology 96(4):558-560 doi:10.1016/s0091-6749(95)70301-2 |
| Auer H, Leskowschek H, Engler J, Leitner G, Wentzel C, Wolkerstorfer W, Schneider R (2007) Epidemiology and nosology of anisakiosis, a rather rare helminthozoonosis in Central Europe - two case reports. Wiener Klinische Wochenschrift 119:106-109 doi:10.1007/s00508-007-0866-4 |
| Autier B, Stephant S, Beusnel C, Sichel P, Belaz S, Gangneux JP, Robert-Gangneux F (2021) Anaphylaxis due to anisakidosis. Clinical Microbiology and Infection 27(1):67-68 doi:10.1016/j.cmi.2020.06.005 |
| Baeza-Trinidad R, Pinilla-Moraza J, Moreno-Medina A (2015) Proximal ileitis secondary to anisakiasis. Acta Clinica Belgica 70(5):387-387 doi:10.1080/17843286.2015.1131963 |
| Bao Pérez F, Álvarez Rubio M, Martí Cabané J (2005) Anisakis simplex sobre ulcus en paciente con Billroth II. Revista Espanola De Enfermedades Digestivas 97(7):532-533 |
| Baptista-Fernandes T, Rodrigues M, Castro I, Paixao P, Pinto-Marques P, Roque L, Belo S, Ferreira PM, Mansinho K, Toscano C (2017) Human gastric hyperinfection by Anisakis simplex: A severe and unusual presentation and a brief review. International Journal of Infectious Diseases 64:38-41 doi:10.1016/j.ijid.2017.08.012 |
| Barbarroja-Escudero J, Rodriguez-Rodriguez M, Sanchez-Gonzalez MJ, Antolin-Amerigo D, Alvarez-Mon M (2013) Anisakis simplex: A new etiological agent of Kounis syndrome. International Journal of Cardiology 167(6):E187-E189 doi:10.1016/j.ijcard.2013.04.058 |
| Barlass U, Rzepczynski A, Naqshband S, Lee S (2019) An uncommon cause of vomiting and abdominal pain in pregnancy. American Journal of Gastroenterology 114:S1490-S1491 doi:10.14309/01.ajg.0000600328.05775.0d |
| Baron L, Branca G, Trombetta C, Punzo E, Quarto F, Speciale G, Barresi V (2014) Intestinal anisakidosis: Histopathological diagnosis findings and differential diagnosis. Pathology Research and Practice 210(11):746-750 doi:10.1016/j.prp.2014.06.022 |
| Barriga J, Salazar F, Barriga E (1999) Anisakiasis: presentación de un caso y revisión de la literatura. Rev Gastroent Perú 19:317-323 |
| Bartra Balcells E, Domenech Calvet J, del Castillo Dejardin D (2021) Intestinal obstruction caused by Anisakis. Revista Espanola De Enfermedades Digestivas 113(7):547-547 doi:10.17235/reed.2020.7409/2020 |
| Beig J, Lane RJ, Lane MR (2019) Gastric anisakiasis: a rare cause of abdominal pain. Internal Medicine Journal 49(1):129-129 doi:10.1111/imj.14194 |
| Bernardo S, Castro-Pocas F (2018) Gastric anisakiasis. Gastrointestinal Endoscopy 88(4):766-766 doi:10.1016/j.gie.2018.06.009 |
| Bhargava D, Raman R, ElAzzouni MZ, Bhargava K, Bhusnurmath B (1996) Anisakiasis of the tonsils. Journal of Laryngology and Otology 110(4):387-388 doi:10.1017/s0022215100133717 |
| Bhat M, Cleland P (2010) Gastric Anisakiasis. Clinical Gastroenterology and Hepatology 8(8):A20 doi:10.1016/j.cgh.2009.12.020 |
| Biondi G, Basili G, Lorenzetti L, Prosperi V, Angrisano C, Gentile V, Goletti O (2008) Acute abdomen due to anisakidosis. Chirurgia italiana 60(4):623-6 |
| Bircher AJ, Gysi B, Zenklusen HR, Aerni R (2000) Eosinophilic oesophagitis associated with recurrent urticaria: is there a worm (Anisakis simplex) in the rose? Schweizerische Medizinische Wochenschrift 130(47):1814-1819 |
| Bolado AG, Gorrino O, Ruiz P, Lecumberri I, Grande D (2003) Intestinal anisakiasis. Radiological diagnosis. Revista Espanola De Enfermedades Digestivas 95(6):443-445 |
| Bong LH, Seon KH, 윤대흥, 조수형 (2013) A Case of Small Bowel Obstruction and Perforation by Anisakiasis. Journal of The Korean Society of Emergency Medicine 24(2):246-249 |
| Bookhout C, Greene K (2019) Chronic epigastric pain associated with gastric anisakidosis. Idcases 15 doi:10.1016/j.idcr.2019.e00515 |
| Bouree P, Zetlaoui J (1989) Spontaneously regressive acute anisakiasis. Presse Medicale 18(38):1885-1885 |
| Brandt LJ (2014) Acute pancreatitis caused by Anisakis. Gastrointestinal Endoscopy 79(4):676-678 doi:10.1016/j.gie.2013.11.026 |
| Brieau B, Rahmi G, Benosman H, Cellier C (2015) Acute dysphagia and odynophagia revealing an unusual case of oesophageal anisakiasis. Digestive and Liver Disease 47(12):E21-E21 doi:10.1016/j.dld.2015.07.047 |
| Brunet J, Pesson B, Royant M, Lemoine J-P, Pfaff AW, Abou-Bacar A, Yera H, Frealle E, Dupouy-Camet J, Merino-Espinosa G, Gomez-Mateos M, Martin-Sanchez J, Candolfi E (2017) Molecular diagnosis of Pseudoterranova decipiens s.s in human, France. Bmc Infectious Diseases 17 doi:10.1186/s12879-017-2493-7 |
| Cabrera R, Del Pilar M, Altamirano T (2004) Anisakidosis a marine parasitic zoonosis: unknown or emerging in Peru? Revista de gastroenterologia del Peru : organo oficial de la Sociedad de Gastroenterologia del Peru 24(4):335-42 |
| Cabrera R, Luna-Pineda MA, Suarez-Ognio L (2003) New case of human infection by a Pseudoterranova decipiens larva (Nematode, Anisakidae) in Peru. Revista de gastroenterologia del Peru : organo oficial de la Sociedad de Gastroenterologia del Peru 23(3):217-20 |
| Cancrini G, Magro G, Giannone G (1997) 1st case of extra-gastrointestinal anisakiasis in a human diagnosed in Italy. Parassitologia 39(1):13-7 |
| Canut Blasco A, Labora Loriz A, de Torre Ramirez JL, Romeo Ramirez JA (1996) Acute gastric anisakiasis caused by insufficient microwave cooking. Medicina Clinica 106(8):317-8 |
| Caramello P, Vitali A, Canta F, Caldana A, Santi F, Caputo A, Lipani F, Balbiano R (2003) Intestinal localization of anisakiasis manifested as acute abdomen. Clinical Microbiology and Infection 9(7):734-737 doi:10.1046/j.1469-0691.2003.00660.x |
| Carbotta G, Laforgia R, Milella M, Sederino MG, Minafra M, Fortarezza F, Piscitelli D, Palasciano N (2016) Small bowel obstruction caused by Anisakis and Meckel's diverticulum: a rare case. Giornale Di Chirurgia 37(6):281-283 doi:10.11138/gchir/2016.37.6.281 |
| Carlin AF, Abeles S, Chin NA, Lin GY, Young M, Vinetz JM (2018) Case Report: A Common Source Outbreak of Anisakidosis in the United States and Postexposure Prophylaxis of Family Collaterals. American Journal of Tropical Medicine and Hygiene 99(5):1219-1221 doi:10.4269/ajtmh.18-0586 |
| Carmo J, Marques S, Bispo M, Serra D (2017) Anisakiasis: a growing cause of abdominal pain! BMJ case reports 2017 doi:10.1136/bcr-2016-218857 |
| Carrascosa MF, Corral Mones J, Salcines-Caviedes JR, Gomez Roman J (2015) A man with unsuspected marine eosinophilic gastritis. Lancet Infectious Diseases 15(2):248-248 doi:10.1016/s1473-3099(14)70892-8 |
| Castillo D C, Martínez C V, Ossandón C F (2003) Anisakiasis en un lactante. Revista chilena de pediatría 74(4):415-416 |
| Cavallero S, Scribano D, D'Amelio S (2016) First case report of invasive pseudoterranoviasis in Italy. Parasitology International 65(5):488-490 doi:10.1016/j.parint.2016.07.003 |
| Centonze A, Capillo S, Mazzei A, Salerno D, Sinopoli D, Prosperi Porta I, Baldassarre E (2021) Acute scrotum in a 8-year-old Italian child caused by extraintestinal anisakiasis in a seaside area. Allergy 76(5):1601-1602 doi:10.1111/all.14779 |
| Cespedes M, Saez A, Rodriguez I, Pinto JM, Rodriguez R (2000) Chronic anisakiasis presenting as a mesenteric mass. Abdominal Imaging 25(5):548-550 doi:10.1007/s002610000089 |
| Cha EJ, Kim JS, Heo SJ (2022) Anisakiasis in Palatine Tonsil. Journal of Craniofacial Surgery 33(7):E692-E694 doi:10.1097/scs.0000000000008580 |
| Chagnon S, El Hajjam M, Laurent V (2007) Cas n° 1. Journal De Radiologie 88(7-8):990-992 doi:10.1016/s0221-0363(07)89911-2 |
| Chikamori F, Kuniyoshi N, Takase Y (2004) Intussusception due to intestinal anisakiasis: a case report. Abdominal Imaging 29(1):39-41 doi:10.1007/s00261-003-0071-5 |
| Cho MH, Lee SJ, Joung HC, Kang JW, Lee KW, Kim YD, Cheon GJ (2012) A case of gastric and colonic submucosal tumors after the removal of 51 Anisakis larvae. The Korean Journal of Medicine 82(4):453-458 |
| Choi JH, Kim E, Park SH, Kim SW (2015) A case of anisakiasis invading the oropharynx. Korean Journal of Otorhinolaryngology Head and Neck Surgery 58(4):284-286 doi:10.3342/kjorl-hns.2015.58.4.284 |
| Choi JW, Park BK, Kim YR, Park SW, Lee SJ, Song SY, Lim BJ, Chung JB (2005) Endoscopic ultrasonographic findings of two cases of parasitic eosinophilic granuloma in the stomach. Clinical Endoscopy 30(5):267-272 |
| Choi SK, Kim CK, Kim SH, Jo DI (2017) Anisakiasis involving the oral mucosa. Archives of craniofacial surgery 18(4):261-263 doi:10.7181/acfs.2017.18.4.261 |
| Choi YI, Park DK, Cho HY, Choi SJ, Chung J-W, Kim KO, Kwon KA, Kim YJ (2019) Adult intussusception caused by colonic anisakis: A case report. World Journal of Clinical Cases 7(17):2536-2541 doi:10.12998/wjcc.v7.i17.2536 |
| Chopra N, Chen CK, Carlson I, Jackson C-C, Mavrogiorgos N (2016) An 11-Year-Old Boy With Sudden-Onset Abdominal Pain. Clinical Infectious Diseases 63(6):844-845 doi:10.1093/cid/ciw414 |
| Cocheton JJ, Gombeaud T, Ferroir JP (1984) Acute duodenal anisakiasis. Presse Medicale 13(12):744-744 |
| Colas Ruiz E, Lasala Gausa MA, Garcia Garcia E, Fernandez Font JM, Martin Cavanna J, Fernandez Cebrian JM (2017) Anisakis and colonic polyp, a rare association. Gastroenterologia Y Hepatologia 40(1):21-23 doi:10.1016/j.gastrohep.2015.10.011 |
| Cortez N, Berzosa M (2020) Anisakiasis mimicking a gastric tumor. American Journal of Gastroenterology 115:S1607-S1607 |
| Couture C, Measures L, Gagnon J, Desbiens C (2003) Human intestinal anisakiosis due to consumption of raw salmon. American Journal of Surgical Pathology 27(8):1167-1172 doi:10.1097/00000478-200308000-00017 |
| Cruchaga S, Pascual J, Munoz F, Guerra A, Ladron de Guevara C (1995) Endoscopic discovery of a worm larva at the base of a gastric ulcer. Enfermedades Infecciosas Y Microbiologia Clinica 13(10):631-2 |
| Cuende E, Audicana MT, Garcia M, Anda M, de Corres LF, Jimenez C, Vesga JC (1998) Rheumatic manifestations in the course of anaphylaxis caused by Anisakis simplex. Clinical and Experimental Rheumatology 16(3):303-304 |
| Cusi Sanchez V, Gonzalez-Cuevas A, Juncosa Morros T, Portus Vinyeta M, Parri Ferrandis F (1999) Anisakiasis in incarcerated epigastric hernia. Anales espanoles de pediatria 50(4):413-4 |
| da Cruz AR, de Sousa Souto PC, Ferrari CKB, Allegretti SM, Arrais-Silva WW (2010) Endoscopic imaging of the first clinical case of anisakidosis in Brazil. Sci Parasitol 11(2):97-100 |
| Dai Z, Kobayashi D (2019) Gastric anisakiasis presenting as a vanishing tumor. Journal of General and Family Medicine 20(4):159-160 doi:10.1002/jgf2.248 |
| D'Amelio S, Mathiopoulos KD, Brandonisio O, Lucarelli G, Doronzo F, Paggi L (1999) Diagnosis of a case of gastric anisakidosis by PCR-based restriction fragment length polymorphism analysis. Parassitologia 41(4):591-3 |
| Daschner A, Alonso-Gomez A, Caballero T, Barranco P, Suarez-De-Parga JM, Lopez-Serrano MC (1998) Gastric anisakiasis: an underestimated cause of acute urticaria and angio-oedema? British Journal of Dermatology 139(5):822-828 |
| Daschner A, Alonso-Gómez A, Mora C, Moreno-Ancillo A, Villanueva R, López-Serrano M (1997) Anisakiasis gastro-alérgica con parasitación masiva. Rev Esp Alergol Inmunol Clin 12(6):370-372 |
| De las Vecillas Sanchez L, Martinez Molina S, Montecchiani V, Drake Perez M, Morchon Miguel E, Rodriguez Fernandez F (2015) Small bowel Anisakiasis. The importance of a detailed clinical history in acute abdomen. Allergy 70:609-609 |
| De Nicola P, Napolitano L, Di Bartolomeo N, Waku M, Innocenti P (2005) Anisakiasis presenting as perforated ulcera of the cecum. Giornale Di Chirurgia 26(10):375-377 |
| de Vera F, Carnicer F, Morales C, Niveiro M (2002) Ileitis with recurrent ascites. Revista Clinica Espanola 202(1):37-38 doi:10.1016/s0014-2565(02)70975-2 |
| De Vincentis F, Caponi A, Mussetto A, Triossi O (2021) Asymptomatic Colonic Anisakiasis. Digestive and Liver Disease 53(5):650-651 doi:10.1016/j.dld.2020.06.029 |
| Deardorff TL, Altman J, Nolan CM (1987) Human anisakiasis - 2 case-reports from the state of Washington. Proceedings of the Helminthological Society of Washington 54(2):274-275 |
| Deardorff TL, Fukumura T, Raybourne RB (1986) Invasive anisakiasis: A case report from Hawaii. Gastroenterology 90(4):1047-1050 doi:10.5555/uri:pii:0016508586908863 |
| Deardorff TL, Kayes SG, Fukumura T (1991) Human anisakiasis transmitted by marine food products. Hawaii medical journal 50(1):9-16 |
| Debat J, Prevost AG, Lefevre P, Anache D, Tahon O (1983) Eosinophil granuloma of the small-intestine and anisakiasis. Gastroenterologie Clinique Et Biologique 7:A76-A76 |
| deCorres LF, Audicana M, DelPozo MD, Munoz D, Fernandez E, Navarro JA, Garcia M, Diez J (1996) Anisakis simplex induces not only anisakiasis: Report on 28 cases of allergy caused by this nematode. Journal of Investigational Allergology and Clinical Immunology 6(5):315-319 |
| Decruyenaere P, Van de Maele B, Hulstaert E, Van Vlierberghe H, Decruyenaere J, Lapeere H (2022) IgE-mediated gastroallergic anisakiasis with eosinophilic oesophagitis: a case report. Acta Clinica Belgica 77(2):396-399 doi:10.1080/17843286.2020.1822627 |
| del Olmo EM, Ibanez AC, de Victoria JMM, Tirao CU (1998) Ileal anisakiasis. Revista Espanola De Enfermedades Digestivas 90(2):120-123 |
| del Olmo Martinez L, Gonzalez de Canales P, Sanjose Gonzalez G (2000) Gastric anisakiasis diagnosed with endoscopy. Anales de medicina interna (Madrid, Spain : 1984) 17(8):429-31 |
| del Pozo V, Arrieta I, Tunon T, Cortegano I, Gomez B, Cardaba B, Gallardo S, Rojo M, Renedo G, Palomino P, Tabar AI, Lahoz C (1999) Immunopathogenesis of human gastrointestinal infection by Anisakis simplex. Journal of Allergy and Clinical Immunology 104(3):637-643 doi:10.1016/s0091-6749(99)70336-2 |
| Demontigny S, Prevot S, Basset D (1991) Gastric anisakiasis cured by delayed endoscopic extraction. Presse Medicale 20(4):180-180 |
| Do K-R, Cho Y-S, Kim H-K, Hwang B-H, Shin E-J, Jeong H-B, Kim S-S, Chae H-S, Choi M-G (2010) Intestinal Helminthic Infections Diagnosed by Colonoscopy in a Regional Hospital during 2001-2008. Korean Journal of Parasitology 48(1):75-78 doi:10.3347/kjp.2010.48.1.75 |
| Doby JM, Masson JM, Babin P (1974) Eosinophilic granuloma of the colon resulting from the larva of the nematode Anisakidae. Bulletin de la Societe de pathologie exotique et de ses filiales 67(5):522-36 |
| Doi R, Inoue K, Gomi T, Sumi S, Yamaki K, Maetani S, Tobe T (1989) A case of anisakiasis as a cause of ileum obstruction. Digestive Surgery 6(4):218-& doi:10.1159/000171928 |
| Dominguez-Ortega J, Alonso-Llamazares A, Rodriguez L, Chamorro M, Robledo T, Bartolome JM, Martinez-Cocera C (2001) Anaphylaxis due to hypersensitivity to Anisakis simplex. International Archives of Allergy and Immunology 125(1):86-88 doi:10.1159/000053801 |
| Dupouy-Camet J, Gay M, Bourgau O, Nouchi A, Leger E, Dei-Cas E (2014) Oesophageal localization: A rare complication of anisakidosis due to Pseudoterranova. Presse Medicale 43(1):81-83 doi:10.1016/j.lpm.2013.01.070 |
| Duran FG, Senent C, deAyala VP, Menchen P, Robles J, Cos E, HernandezAlbujar A (1996) Endoscopic diagnosis of a skin rush. Revista Espanola De Enfermedades Digestivas 88(10):713-714 |
| Eguia A, Aguirre JM, Echevarria MA, Martinez-Conde R, Ponton J (2003) Gingivostomatitis after eating fish parasitized by Anisakis simplex: A case report. Oral Surgery Oral Medicine Oral Pathology Oral Radiology and Endodontology 96(4):437-440 doi:10.1016/s1079-2104(03)00264-6 |
| Eskesen A, Strand EA, Andersen SN, Rosseland A, Hellum KB, Strand OA (2001) Anisakiasis presenting as an obstructive duodenal tumor. A Scandinavian case. Scandinavian Journal of Infectious Diseases 33(1):75-76 |
| Esteve C, Resano A, Diaz-Tejeiro P, Fernandez-Benitez M (2000) Eosinophilic gastritis due to Anisakis: a case report. Allergologia Et Immunopathologia 28(1):21-3 |
| Fabresse FX, Essioux H, Meyran M, Larroque P, Celton H (1984) Polyarthritis in anisakiasis - 1st case. Presse Medicale 13(16):1004-1004 |
| Fernandez Salazar LI, Guantes de Vigo B, Herreros Rodriguez J, Abril Vega C, Calabia del Campo J, de la Calle Valverde F, Velayos Jimenez B, Gonzalez Hernandez JM (2010) Another multiple gastric anisakiasis case. Revista Espanola De Enfermedades Digestivas 102(1):60-61 |
| Filauro M, Rollandi GA, Cassola G, Quilici P, Angelini G, Belli F, Boccardo C (2011) Gastrointestinal bleeding due to suspected anisakiasis: challenging differential diagnosis for a rare disease. Updates in Surgery-Italy 63(3):213-217 doi:10.1007/s13304-011-0055-x |
| Fuchizaki U, Nishikawa M (2016) Gastric Anisakiasis. New England Journal of Medicine 375(7):E11-E11 doi:10.1056/NEJMicm1509451 |
| Fujii M, Okabayashi Y, Tateiwa S, Okamoto Y, Sakai T, Ochi F, Sugano M, Oshiro K (2002) Vanishing gastric tumor. Gastrointestinal Endoscopy 56(3):421-421 doi:10.1067/mge.2002.126701 |
| Fujikawa H, Kuwai T, Yamaguchi T, Miura R, Sumida Y, Takasago T, Miyasako Y, Nishimura T, Iio S, Imagawa H, Yamaguchi A, Kouno H, Kohno H (2018) Gastric and enteric anisakiasis successfully treated with Gastrografin therapy: A case report. World Journal of Gastrointestinal Endoscopy 10(3):69-73 doi:10.4253/wjge.v10.i3.69 |
| Fujisawa K, Matsumoto T, Yoshimura R, Ayabe S, Tominaga M (2001) Endoscopic finding of a large vanishing tumor. Endoscopy 33(9):820-820 doi:10.1055/s-2001-16517 |
| Fukita Y, Asaki T, Katakura Y (2014) Some Like It Raw: An Unwanted Result of a Sushi Meal. Gastroenterology 146(5):E8-E9 doi:10.1053/j.gastro.2013.12.040 |
| Fukui S, Matsuo T, Mori N. Palatine tonsillar infection by Pseudoterranova azarasi. Am J Trop Med Hyg 2020;103:8. |
| Fung BM, Ostrzega N, Tabibian JH (2018) New-Onset Undulatory Abdominal Pain: Revelations of Timely Endoscopic Intervention. Gastroenterology 155(4):985-987 doi:10.1053/j.gastro.2018.03.051 |
| Furukawa K, Yoshida K, Nojiri T, Ogawa M, Kohno S, Yanaga K (2014) Adult intussusception caused by Meckel's diverticulum complicated by anisakiasis of the small intestine: report of a case. Clinical Journal of Gastroenterology 7(4):316-319 doi:10.1007/s12328-014-0502-6 |
| Gallo R, Cecchi F, Parodi A (2012) Intractable chronic pruritus as the only manifestation of IgE hypersensitivity to Anisakis. Journal of the American Academy of Dermatology 67(6):E261-E261 doi:10.1016/j.jaad.2012.03.026 |
| Garcia Garcia JM, Romero Arauzo MJ (2004) Angina-like chest pain due to gastric anisakiasis. Anales de medicina interna (Madrid, Spain : 1984) 21(4):185-6 |
| Garcia-Labairu C, Alonso-Martinez JL, Martinez-Echeverria A, Rubio-Vela T, Zozaya-Urmeneta JM (1999) Asymptomatic gastroduodenal anisakiasis as the cause of anaphylaxis. European Journal of Gastroenterology & Hepatology 11(7):785-787 doi:10.1097/00042737-199907000-00018 |
| Ghimire R, Urban C, Gervacio B, Tong J, Kim S, Segal-Maurer S (2016) Gastrointestinal: Upper gastrointestinal bleeding due to an unusual parasite. Journal of Gastroenterology and Hepatology 31(8):1383-1383 doi:10.1111/jgh.13411 |
| Gill CJ, Hamer DH (2005) An uninvited dinner guest. Clinical Infectious Diseases 41(12):1764-+ |
| Godeau P, Danis M, Bouchareine A, Nozais JP (1985) Anisakiasis - an unusual cause of segmental edema. Presse Medicale 14(22):1246-1247 |
| Goldfain D, Potet F (1984) Gastric anisakiasis - endoscopic diagnosis and treatment. Presse Medicale 13(42):2586-2586 |
| Gonzalez-Bertolin B, Hernanz-Ruiz N, Perez-Tanoira R, Perteguer-Prieto MJ (2021) Colonic anisakiasis, an infrequent case molecularly characterized by PCR-RFLP. Enfermedades infecciosas y microbiologia clinica (English ed) 39(6):308-309 doi:10.1016/j.eimce.2021.04.001 |
| Hajjar R, Chakravarti A, Malaekah H, Schwenter F, Lemieux C, Maietta A, Sebajang H (2020) Anisakiasis in a Canadian patient with incarcerated epigastric hernia. Idcases 20 doi:10.1016/j.idcr.2020.e00715 |
| Hamada K, Uedo N, Tomita Y, Iishi H (2016) A bleeding gastric ulcer caused by anisakiasis. Annals of Gastroenterology 29(3):378-378 doi:10.20524/aog.2016.0039 |
| HAMANAKA K, SODEYAMA H, TAKAHASHI K, NISHIO A, NAKATA S, ONUMA H (2001) A case of intussusception caused by anisakiasis of the ileocecal resion. Nihon Rinsho Geka Gakkai Zasshi (Journal of Japan Surgical Association) 62(7):1668-1671 |
| Hamdan M, Wagner J (1992) Pseudotumoral anisakiasis of the stomach revealed by digestive bleeding - a case-report and review of the literature. Semaine Des Hopitaux 68(19):559-561 |
| Hara Y, Uruma T, Morishima Y, Hirai Y (2019) 'Tingling throat syndrome' as asymptomatic anisakiasis following conveyor belt sushi consumption in Tokyo. International Journal of Infectious Diseases 82:102-103 doi:10.1016/j.ijid.2019.03.019 |
| Hashimoto R, Chonan A (2016) Gastric Anisakiasis with a Gastric Ulcer. Internal Medicine 55(24):3681-3681 doi:10.2169/internalmedicine.55.7433 |
| Hashimoto R, Matsuda T, Nakahori M (2017) Small bowel anisakiasis detected by capsule endoscopy. Digestive Endoscopy 29(1):126-127 doi:10.1111/den.12738 |
| Hashimoto Y, Nagao G, Matsuoka H, Abe N, Yanagida O, Masaki T, Moti, Sugiyama M. Report of a case: small bowel intussusception caused by Anisakiasis (in Japanese). J Jpn Soc Surg Infect. 2011;8:741–5. |
| Hayashi N, Furukawa M, Nakata T, Kusano T, Lin Y, Tashiro K, Watabe S, Suga K, Miyazaki K, Fujii H (1989) A case of intestinal anisakiasis associated with intussusceptions of the small intestine. Iryo 43:753-756 |
| Hernandez-Prera JC, Polydorides AD (2012) Anisakidosis of the sigmoid colon disguising as metastatic carcinoma: A case report and review of the literature. Pathology Research and Practice 208(7):433-435 doi:10.1016/j.prp.2012.05.004 |
| Herranz Bachiller MT, Atienza Sanchez R, Barrio Andres J, Alcaide Suarez N, Ruiz-Zorrilla R, Sancho del Val L, de la Serna Higuera C, Perez Miranda M (2012) Colonic polyp secondary to Anisakis simplex. Revista Espanola De Enfermedades Digestivas 104(10):554-555 doi:10.4321/s1130-01082012001000011 |
| Hibi Y, Ogata J, Mimuro A, Ito K, Hakamada Y (2009) A case of small intestinal intussusceptions caused by anisakidosis. Japanese J Gastroenterol Surg 42:669-673 |
| Higashi M, Tanaka K, Kitada T, Nakatake K, Tsuji M (1988) Anisakiasis confirmed by radiography of the large-intestine. Gastrointestinal Radiology 13(1):85-86 doi:10.1007/bf01889030 |
| Hijikata N, Takayanagi N, Sugita Y, Kawabata Y (2010) Dyspnoea, fever, patchy ground-glass opacities and intermittent severe epigastralgia. Thorax 65(10):920-890, 920 doi:10.1136/thx.2010.136655 |
| Hiramatsu K, Kamiyamamoto S, Ogino H, Satomura Y, Konishi K, Miwa A, Demachi H, Noda Y (2004) A case of acute gastric anisakiasis presenting with malignant tumor-like features: A large gastric vanishing tumor accompanied by local lymph node swelling. Digestive Diseases and Sciences 49(6):965-969 doi:10.1023/B:DDAS.0000034555.55739.3c |
| Hiramoto JT, Tokeshi J (1991) Anisakiasis in Hawaii: a radiological diagnosis. Hawaii medical journal 50(6):202-3 |
| HIRANO S, ANAMI Y (1998) A case of intussusception caused by anisakiasis of the small intestine. Nihon Rinsho Geka Gakkai Zasshi (Journal of Japan Surgical Association) 59(2):408-411 |
| Hirosawa T, Sakamoto T, Shimizu T (2020) Gastric Anisakiasis. American Journal of the Medical Sciences 360(3):318-319 |
| Hoang C, Garin Y, Ichou J, Lecharpentier Y (1985) Jejunal anisakiasis with intestinal-obstruction. Gastroenterologie Clinique Et Biologique 9(11):847-848 |
| Hokama A, Gakiya I, Miyagi T, Fukuchi J, Kinjo F, Saito A (2005) Gastrointestinal: Acute gastric anisakiasis. Journal of Gastroenterology and Hepatology 20(7):1121-1121 doi:10.1111/j.1440-1746.2005.04009.x |
| Hokama A, Oshiro T, Tomisato K, Nakamatsu G, Tameda S, Fujita J (2019) Gastric anisakidosis: an unfavorable taste of sushi. Polish Archives of Internal Medicine-Polskie Archiwum Medycyny Wewnetrznej 129(7-8):547-548 doi:10.20452/pamw.14838 |
| Holzgreve H (2018) Der Parasit aus dem Fischgericht. MMW Fortschritte der Medizin 160(12):35-35 doi:10.1007/s15006-018-0700-0 |
| Hope W, Smith-Chakmakova F, Snyder J (2020) Case of anisakiasis presenting as an Amyand hernia. BMJ case reports 13(7) doi:10.1136/bcr-2020-234822 |
| Horwitz MA, Hughes JM (1976) Outbreaks of food-borne disease in United-States, 1974. Journal of Infectious Diseases 134(3):306-312 doi:10.1093/infdis/134.3.306 |
| Hoshino C, Narita M (2011) Anisakis simplex-induced anaphylaxis. Journal of Infection and Chemotherapy 17(4):544-546 doi:10.1007/s10156-011-0209-2 |
| Hsiu JG, Gamsey AJ, Ives CE, Damato NA, Hiller AN (1986) Gastric anisakiasis - report of a case with clinical, endoscopic, and histological-findings. American Journal of Gastroenterology 81(12):1185-1187 |
| Humphrey F, Connolly S (2016) A Cautionary Tale for Sushi Lovers. American Journal of Gastroenterology 111:S1103-S1104 doi:10.14309/00000434-201610001-02280 |
| Hwang D, Park SI, Pack SC, Lee KS, Choi SK, Kang H, Park CW, Lee S (2012) A case of duodenal anisakiasis with duodenal ulcer. Chonnam medical journal 48(1):73-5 doi:10.4068/cmj.2012.48.1.73 |
| Ido K, Yuasa H, Ide M, Kimura K, Toshimitsu K, Suzuki T (1998) Sonographic diagnosis of small intestinal anisakiasis. Journal of Clinical Ultrasound 26(3):125-130 doi:10.1002/(sici)1097-0096(199803/04)26:3<125::Aid-jcu3>3.3.Co;2-w |
| Iglesias-Hidalgo A, Mane-Ruiz N, Mendaza-Beltran P (2003) Acute abdomen due to Anisakis simplex. Enfermedades Infecciosas Y Microbiologia Clinica 21(6):323-324 doi:10.1157/13048587 |
| Ikeda K, Kumashiro R, Kifune T (1989) 9 cases of acute gastric anisakiasis. Gastrointestinal Endoscopy 35(4):304-308 doi:10.1016/s0016-5107(89)72797-8 |
| Ikegami K, Hirose Y, Yoneyama O (2018) An Unusual Cause of Severe Epigastric Pain. Gastroenterology 154(6):E7-E8 doi:10.1053/j.gastro.2017.08.021 |
| Ikuta R, Kitazawa H, Matsuda K, Tashiro M (2016) Anisakiasis of the Anorectum. Internal Medicine 55(17):2513-2514 doi:10.2169/internalmedicine.55.6918 |
| Ildefonso C, Rodriguez I, Perez-Holanda S, Fernandez A, Llera JM, Mendez B, Granero J (2005) Acute intestinal anisakiasis. Cirugia espanola 78(1):59-60 doi:10.1016/s0009-739x(05)70889-7 |
| Imanishi M, Sato M, Tokuda Y (2012) Endoscopic capture of Anisakis larva (a video demonstration). BMJ case reports 2012 doi:10.1136/bcr.03.2012.6068 |
| Ishida M, Harada A, Egawa S, Watabe S, Ebina N, Unno M (2007) Three successive cases of enteric Anisakiasis. Digestive Surgery 24(3):228-231 doi:10.1159/000103325 |
| Ishiguro A, Uno Y, Ishiguro Y, Sakuraba H, Munakata A (2001) Anisakiasis of the ileocecal valve. Gastrointestinal Endoscopy 53(6):677-679 doi:10.1067/mge.2001.112715 |
| Ishii N, Matsuda M, Setoyama T, Suzuki S, Uchida S, Uemura M, Iizuka Y, Fukuda K, Horiki N, Fujita Y (2009) Anisakiasis and vanishing tumor of the cecum. Endoscopy 41:E226-E227 doi:10.1055/s-0029-1214923 |
| Ito Y, Ikematsu Y, Yuzawa H, Nishiwaki Y, Kida H, Waki S, Uchimura M, Ozawa T, Iwaoka T, Kanematsu T (2007) Chronic gastric anisakiasis presenting as pneumoperitoneum. Asian Journal of Surgery 30(1):67-71 doi:10.1016/s1015-9584(09)60131-7 |
| Iwakami S, Kawakami K, Kawaura Y (1995) A case of intussusception caused by anisakiasis of small intestine. Jpn J Gastroenterol Surg 28:2037-2041 |
| Jaechun L, 김수희, 범종욱, 김성현, 조재민, 송현주 (2012) A Case of Anaphylaxis Caused by Acute Gastric Anisakiasis. 32(4):276-279 |
| Jang S-t, Choi I-j, Kim W-t, Lee H, Lee S-w, Kang S-b, Nam S-w, Lee D-s (2006) A case of rectal anisakiasis. Clinical Endoscopy 32(2):156-159 |
| Jofre M L, Neira O P, Noemi H I, Cerva C JL (2008) Pseudoterranovosis and sushi. Revista Chilena De Infectologia 25(3):200-205 doi:10.4067/s0716-10182008000300010 |
| Jong Hyun Kim, Hwang JU, Kim SH, Kyu Hak Kim, Kang SJ, Kim KS, Hur JH, Choi SH (2006) A case of anisakiasis concurrently invading esophagus and stomach, and another case of esophageal anisakiasis. Clinical Endoscopy 32(2):116-119 |
| Joo DC, Kim GH, Lee MW (2021) Acute Anisakiasis at the Esophagogastric Junction Mimicking Angina Pectoris. Korean Journal of Helicobacter Upper Gastrointestinal Research 21(2):161-164 doi:10.7704/kjhugr.2021.0008 |
| Joo SK, Kim JW, Kim BG, Kim W, Lee JK, Lee KL (2019) Clinical and Endoscopic Features of Colonic Anisakiasis in Korea. Korean Journal of Parasitology 57(4):411-+ doi:10.3347/kjp.2019.57.4.411 |
| Jordan-Domingo M, Lahoza-Perez MC, Perez-Aradros-Hernandez C, Monzon-Ballarin S, Saenz-Abad D (2017) Endoscopic treatment of a recurrent urticaria. Semergen 43(2):164-167 doi:10.1016/j.semerg.2016.04.014 |
| Jung CW, Cho DH, Yoo GJ, Shim SG (2009) A case of colonic anisakiasis mimicking submucosal tumor. Journal of Gastroenterology and Hepatology 24:A71-A71 |
| Jurado-Palomo J, Lopez-Serrano MC, Moneo I (2010) Multiple Acute Parasitization by Anisakis simplex. Journal of Investigational Allergology and Clinical Immunology 20(5):437-441 |
| Juric I, Pogorelic Z, Despot R, Mrklic I (2013) Unusual cause of small intestine obstruction in a child Small intestine anisakiasis: report of a case. Scottish Medical Journal 58(1):E32-E36 doi:10.1177/0036933012474616 |
| Just PA, Meatchi T, Elouaret Y, Badoual C, Couchon S (2008) Anisakidosis: an emmergent disease. Gastroenterologie Clinique Et Biologique 32(8-9):782-784 doi:10.1016/j.gcb.2008.01.025 |
| Kagei N, Isogaki H (1992) A case of abdominal syndrome caused by the presence of a large number of Anisakis larvae. International Journal for Parasitology 22(2):251-253 |
| Kaila V, Rosenstein D (2018) Incidental Jejunal Anisakiasis in a Patient Presenting With Small Bowel Obstruction. American Journal of Gastroenterology 113:S1393-S1393 |
| Kajihara Y (2018) A young man with acute onset epigastric pain after the ingestion of Japanese sushi. European Journal of Internal Medicine 57:E3-E4 doi:10.1016/j.ejim.2018.02.032 |
| Kajihara Y (2022) Vanishing tumor of the stomach. European Journal of Internal Medicine 101:110-111 doi:10.1016/j.ejim.2022.03.022 |
| Kanematsu M, Hoshi H (1997) Benefit of immediate endoscopy after radiography for anisakiasis. American Journal of Roentgenology 168(4):1114-1115 doi:10.2214/ajr.168.4.9124130 |
| Kang DB, Oh JT, Park WC, Lee JK (2010) Small bowel obstruction caused by acute invasive enteric anisakiasis. The Korean journal of gastroenterology = Taehan Sohwagi Hakhoe chi 56(3):192-5 |
| Kang DB, Park WC, Lee JK (2014) Chronic gastric anisakiasis provoking a bleeding gastric ulcer. Annals of Surgical Treatment and Research 86(5):270-273 doi:10.4174/astr.2014.86.5.270 |
| Kang WH, Kim KS, Lee SH, Park YW (2019) Gastric anisakiasis after eating raw salmon. Digestive and Liver Disease 51(4):602-602 doi:10.1016/j.dld.2018.10.021 |
| Kanisawa Y, Kawanishi N, Hisai H, Araya H (2000) Colonic anisakiasis: An unusual cause of intussusception. Endoscopy 32(9):S55-S55 |
| Kapral C, Haditsch M, Wewalka F, Schatzlmayr W, Lenz K, Auer H (2009) The First Case of Anisakiasis Acquired in Austria. Zeitschrift Fur Gastroenterologie 47(10):1059-1061 doi:10.1055/s-0028-1109468 |
| Karmanova IV, Plashkova VV, Nechaeva OI, Gubina VV (2002) A case of human anisakiasis in Kamchatka. Meditsinskaia parazitologiia i parazitarnye bolezni(2):32-3 |
| Kasuga K, Tanaka H, Hosaka H, Uraoka T (2022) Anisakis with a Gastric Ulcer after Endoscopic Resection. Internal Medicine 61(19):2981-2982 doi:10.2169/internalmedicine.9278-21 |
| Kates S, Wright KA, Wright R (1973) Case of human infection with cod nematode Phocanema sp. American Journal of Tropical Medicine and Hygiene 22(5):606-608 doi:10.4269/ajtmh.1973.22.606 |
| Kawada A, Sasaki S, Iwasaki T, Kojima K, Nakayama M, Uchida K, et al. A case of ileal anisakiasis in which the anisakis larva was removed by endoscopy [in Japanese]. Kochi Red Cross Hospital Igaku Zasshi. 2015;20:15–8. |
| kawadKawada A, Sasaki S, Iwasaki T, Kojima K, Nakayama M, Uchida K ea (2015) A case of ileal anisakiasis in which the anisakis larva was removed by endoscopy [in Japanese]. Kochi Red Cross Hospital Igaku Zasshi 20:15–18 |
| Kawanishi K, Ikeda Y, Furotani M, Tsuboi S, Kanno T, Niwa T, Nagaoka T, Tabata Y, Kitano M (2021) Fifty-millimeter abscess in the ileum caused by perforation from anisakiasis successfully treated with conservative therapy without drainage. Oxford Medical Case Reports(6) doi:10.1093/omcr/omab033 |
| Kawashima K, Fujiwara T, Katakura K, Gunji N, Yokokawa A, Sakamoto A, Hikichi T, Kono K, Ohira H (2019) Anisakiasis in the small intestine with excessive bleeding that was difficult to diagnose endoscopically. Internal Medicine 58(1):63-66 doi:10.2169/internalmedicine.1482-18 |
| Khan MQ, Williams J (2016) Anisakidosis: a fortuitous mimicker of gastrointestinal malignancy. BMJ case reports 2016 doi:10.1136/bcr-2016-216164; Khan MQ, Williams J (2015) Anisakidosis: A Fortuitous Mimicker of Gastrointestinal Malignancy. American Journal of Gastroenterology 110:S547-S547 doi:10.14309/00000434-201510001-01245 |
| Kim BH, Park CU, Lee JH, Yeom SM, Chae DY, Kim SP, Jeon WJ, Lee GH, Kim HD, Im JY (2004) A case of anisakiasis concurrently invading the stomach, ileocecal valve and transverse colon. Clinical Endoscopy 28(1):43-46 |
| Kim BH, Park HU, Park SK, Jeon SM, Jung CW, Son CM, Choi HW, Kim HS (2018) Anisakiasis Induced Segmental Jejunum Obstruction. The Korean journal of gastroenterology = Taehan Sohwagi Hakhoe chi 72(1):33-36 doi:10.4166/kjg.2018.72.1.33 |
| Kim CH, Chung BS, Moon YI, Chun SH (1971) A case report on human infection with Anisakis sp. in Korea. Kisaengch'unghak chapchi The Korean journal of parasitology 9(1):39-43 |
| Kim HJ, Park C, Cho SY (1997) A case of extragastrointestinal anisakiasis involving a mesocolic lymph node. The Korean journal of parasitology 35(1):63-6 |
| Kim H-S, Kwon S, 김문영, 박동훈, 최윤종, 백순구, 이동기 (2002) A case of anisakiasis diagnosed after partial resection of ileum due to eosinophilic ascites and ileal abscess. Clinical Endoscopy 25(6):457-460 |
| Kim J, Mitsui T, Asada Y (1990) A case report of parasitic eosinophilic granuloma of the ileocecal valve causing intussusception. J Clin Surg 45:1825-1828 |
| Kim JH, Kim H-J, Lin CD, 홍성숙, 박성태, 장윤우, 권귀향, 황정화 (2009) Duodenal Anisakiasis Presenting as Bowel Obstruction and Fistula Formation: A Case Report. Journal of the Korean Society of Radiology (JKSR) 60(6):419-422 |
| Kim JW, Lee YJ, Jung GM, Cho YK, Ju MJ, Cho JW, Lee YU (2007) A case of chronic gastric eosinophilic abscess treated by endoscopic submucosal dissection. Clinical Endoscopy 35(1):33-37 |
| Kim LS, Lee YH, Kim S, Park HR, Cho SY (1991) A case of anisakiasis causing intestinal obstruction. Kisaengch'unghak chapchi The Korean journal of parasitology 29(1):93-6 |
| KIM S-G, JO Y-J, PARK Y-S, KIM S-H, SONG M-H, LEE H-H, KIM J-S, RYOU J-W, JOO J-E, KIM D-H (2006) Four cases of gastric submucosal mass suspected as anisakiasis. The Korean journal of parasitology 44(1):81-86 |
| Kim SH, Kim HU, Lee J (2006) A case of gastroallergic anisakiasis. The Korean Journal of Medicine 70(1):111-116 |
| Kim SH, Park CW, Kim SK, Won S, Park WK, Kim HR, Nam KW, Lee GS (2013) A Case of Anisakiasis Invading the Stomach and the Colon at the Same Time after Eating Anchovies. Clinical Endoscopy 46(3):293-296 doi:10.5946/ce.2013.46.3.293 |
| Kim SJ, Kim JW, Kim KJ, Heo C, Kim Sw, Han SP, Seo YH, Do JH, Kim jG, Park SM (2006) Two cases of colon anisakiasis: Asymphtomatic cecal anisakiasis and ascending colon anisakiasis detected one month later after infestation. Clinical Endoscopy 33(2):116-120 |
| Kim YH, Choi WB, Lee SC, Choi HW (2006) Three cases of colonic anisakiasis. Clinical Endoscopy 33(4):239-243 |
| Kinoshita Y, Fujimoto K, Lee M, Shinohara R, Kobayashi Y, Kawana S, Saeki H (2014) Two cases of allergies due to Anisakis simplex, positive to specific IgE for Ani s 12 allergen. Arerugi = [Allergy] 63(10):1348-52 |
| Kita R, Hashida H, Uryuhara K, Kaihara S (2019) Hepatic anisakiasis mimicking metastatic liver tumour. International Journal of Surgery Case Reports 60:209-212 doi:10.1016/j.ijscr.2019.06.010 |
| Kliks MM (1983) Anisakiasis in the western United-states - 4 new case reports from California. American Journal of Tropical Medicine and Hygiene 32(3):526-532 doi:10.4269/ajtmh.1983.32.526 |
| Knofler H, Schweisinger G (1979) Anisakiasis. Zentralblatt Fur Chirurgie 104(6):405-408 |
| Kobayashi A, Tsuji M, Wilbur DL (1985) Probable pulmonary anisakiasis accompanying pleural effusion. American Journal of Tropical Medicine and Hygiene 34(2):310-313 doi:10.4269/ajtmh.1985.34.310 |
| Kojima G, Usuki S, Mizokami K, Tanabe M, Machi J (2013) Intestinal anisakiasis as a rare cause of small bowel obstruction. American Journal of Emergency Medicine 31(9) doi:10.1016/j.ajem.2013.05.015 |
| Kojima H (2022) Intestinal anisakiasis with small bowel obstruction following recurrent gastric anisakiasis. The American journal of the medical sciences doi:10.1016/j.amjms.2022.09.013 |
| Kojima Y, Takeuchi T, Egashira Y, Higuchi K (2017) A Case of Eosinophilic Granuloma Presenting as a Small Submucosal Tumor. Internal Medicine 56(9):1115-1116 doi:10.2169/internalmedicine.56.7734 |
| Kolodziejczyk L, Szostakowska B, Sobecka E, Szczucki K, Stankiewicz K (2020) First case of human anisakiasis in Poland. Parasitology International 76 doi:10.1016/j.parint.2020.102073 |
| Kondo T (2018) Woe sushi: gastric anisakiasis. Lancet 392(10155):1340-1340 doi:10.1016/s0140-6736(18)32276-1 |
| Kondo T, Terada K (2016) Unexpected cause of urticaria. Medical Journal of Australia 204(7) doi:10.5694/mja15.01343 |
| Kowalewska-Grochowska K, Quinn J, Perry I, Sherbaniuk R (1989) A case of anisakiasis--Alberta. Canada diseases weekly report = Rapport hebdomadaire des maladies au Canada 15(44):221-3 |
| Kumagai K, Hirose Y, Yoshida S (2018) Images in emergency medicine, Anisakiasis of the tongue. Annals of Emergency Medicine 72(6):E121-E122 doi:10.1016/j.annemergmed.2018.06.028 |
| Kumagai M (2006) An unusual body in the soft palate. The Nihon University Journal of Medicine 48:79-81 |
| Kwak SY, Yoon Y-H (2012) Laryngeal Anisakiasis: An Unusual Cause of Foreign-Body Sensation in the Throat. Otolaryngology-Head and Neck Surgery 147(3):588-589 doi:10.1177/0194599812443512 |
| Kwee H, Sautter R (1987) Anisakiasis. American Family Physician 36(2):137-140 |
| Kwon JH, Uhm JH, Chung KS (2004) A case of gastric anisakiasis with recurrent abdominal pain in a Child. Pediatric Gastroenterology, Hepatology & Nutrition 7(1):74-77 |
| Lalchandani UR, Weadock WJ, Brady GF, Wasnik AP (2018) Imaging in gastric anisakiasis. Clinical Imaging 50:286-288 doi:10.1016/j.clinimag.2018.04.018 |
| Lee C, Choi J, Kim J (2008) Intestinal obstruction caused by anisakiasis. Annals of Surgical Treatment and Research 74(2):154-156 |
| Lee JI, Choi MG, Kim SW, Lim CH, Kim JS, Cho YK, Lee IS, Choi KY, Chung IS (2011) A case report of anisakiasis developed on ileocecal valve area. Journal of Gastroenterology and Hepatology 26:124-125 |
| Lewis R, Shore JH (1985) Anisakiasis in the United Kingdom. Lancet 2(8462):1019-1019 |
| Li S-W, Shiao S-H, Weng S-C, Liu T-H, Su K-E, Chen C-C (2015) A case of human infection with Anisakis simplex in Taiwan. Gastrointestinal Endoscopy 82(4):757-758 doi:10.1016/j.gie.2015.03.1983 |
| Lichtenfels JR, Brancato FP (1976) Anisakid larva from throat of an Alaskan eskimo. American Journal of Tropical Medicine and Hygiene 25(5):691-693 doi:10.4269/ajtmh.1976.25.691 |
| Lim SR, Hong Sn, Kim DS (2003) An investigation of instance infected after eating a slice of raw fish of Astroconger myriaster. Korean Journal of Clinical Laboratory Science 35(2):86-89 |
| Lock G, Ehresmann J, Joentvedt E (2008) Severe segmental colitis in anisakiasis: Unusual manifestation of a rare infection in Germany. Deutsche Medizinische Wochenschrift 133(36):1779-1782 doi:10.1055/s-0028-1082811 |
| Lopez-Velez R, Garcia A, Barros C, Manzarbeitia F, Onate JM (1992) Anisakiasis in Spain. Report of 3 cases. Enfermedades Infecciosas Y Microbiologia Clinica 10(3):158-61 |
| Lorenz G, Warzok R (1988) Intestinal anisakiasis (infestation by herring worms - a study of 8 acute cases. Pathologe 9(4):199-203 |
| Louredo Mendez AM, Rodriguez Sanz MB, Alonso Poza A (2006) Image of the month. Intestinal anisakiasis. Cirugia espanola 80(1):52-52 |
| Lucas SB, Cruse JP, Lewis AAM (1985) Anisakiasis in the United Kingdom. Lancet 2(8459):843-844 |
| Ludovisi A, Trotta D, Illiceto MT, Mazzochetti C, Lizzi M, Arico M (2022) Hypereosinophilia, Abdominal Pain, and Diarrhea: Anisakiasis, a Difficult Diagnosis in a Child. Pediatric Infectious Disease Journal 41(8):E343-E344 doi:10.1097/inf.0000000000003567 |
| Machi T, Okino S, Saito Y, Horita Y, Taguchi T, Nakazawa T, Nakamura Y, Hirai H, Miyamori H, Kitagawa S (1997) Severe chest pain due to gastric anisakiasis. Internal Medicine 36(1):28-30 doi:10.2169/internalmedicine.36.28 |
| Madi L, Ali M, Legace-Wiens P, Duerksen DR (2013) Gastrointestinal manifestations and management of anisakiasis. Canadian Journal of Gastroenterology and Hepatology 27(3):126-127 doi:10.1155/2013/427982 |
| Maggi P, Caputi-Iambrenghi O, Scardigno A, Scoppetta L, Saracino A, Valente M, Pastore G, Angarano G (2000) Gastrointestinal infection due to Anisakis simplex in southern Italy. European Journal of Epidemiology 16(1):75-78 doi:10.1023/a:1007617002972 |
| Magnaval JF, Berry A, Nadrigny M (2002) Anaphylactic shock revealing anisakiasis. Presse Medicale 31(28):1309-1311 |
| Manabe S, Takahashi S, Yasuoka T, Endo Y, Usui F, Yamaguchi T, Matsuyama T, Hirata I (2017) A case of small intestinal intussusception caused by anisakiasis diagnosed and treated by upper gastrointestinal endoscopy. Nihon Shokakibyo Gakkai zasshi = The Japanese journal of gastro-enterology 114(8):1460-1466 doi:10.11405/nisshoshi.114.1460 |
| Mannami T, Fujiwara N, Ikeda G, Wakatsuki T, Fukumoto Y, Furutachi Si, Shimizu Si (2021) Esophageal anisakiasis observed using magnifying endoscopy with narrow-band imaging. Endoscopy 53(03):E83-E84 doi:10.1055/a-1195-2273 |
| Martinez-Acitores de la Mata D, Arrubla Gamboa A, Albeniz Arbizu E, Larrea Ramirez A (2020) Endoscopic submucosal dissection of colonic anisakiasis. Revista Espanola De Enfermedades Digestivas 112(10):807-808 doi:10.17235/reed.2020.6748/2019 |
| Martinez-Ubieto F, Bueno-Delgado A, Jimenez-Bernado T, Pilar Santero-Ramirez M, Arribas-del Amo D, Martinez-Ubieto J (2013) Acute abdomen caused by eosinophilic enteritis: on purpose of six observations. Cirugia Y Cirujanos 81(3):237-241 |
| Marzocca G, Rocchi B, Lo Gatto M, Polito S, Varrone F, Caputo E, Sorbellini F (2009) Acute abdomen by Anisakiasis and globalization. Annali Italiani Di Chirurgia 80(1):65-68 |
| Masui N, Fujima N, Hasegawa T, Kigawa S, Kagei N, Nagashima K, Shimizu Y (2006) Small bowel strangulation caused by parasitic peritoneal strand. Pathology International 56(6):345-349 doi:10.1111/j.1440-1827.2006.01970.x |
| Matsui S, Uraoka T, Hasegawa H, Kitagawa Y (2015) A case of asymptomatic incidental live anisakid worm infestation on a large rectal polyp. BMJ case reports 2015 doi:10.1136/bcr-2014-208708 |
| Matsuoka H, Nakama T, Kisanuki H, Uno H, Tachibana N, Tsubouchi H, Horii Y, Nawa Y (1994) A case-report of serologically diagnosed pulmonary anisakiasis with pleural effusion and multiple lesions. American Journal of Tropical Medicine and Hygiene 51(6):819-822 doi:10.4269/ajtmh.1994.51.819 |
| Matsushita M, Kobayashi K, Oba K, Nishiyama R, Seki A, Kawata Y, Sakai H, Kobayashi Y (2007) Human small intestinal anisakiasis due to consumption of raw sardines. American Journal of Gastroenterology 102:S279-S280 doi:10.14309/00000434-200709002-00446 |
| Matsuura H, Jinno H (2018) Colonic Anisakiasis. American Journal of Medicine 131(5):E203-E203 doi:10.1016/j.amjmed.2017.11.029 |
| Matsuura H, Moritou Y (2017) Gastric anisakiasis. Qjm-an International Journal of Medicine 110(4):251-251 doi:10.1093/qjmed/hcx009 |
| Mattiucci S, Fazii P, De Rosa A, Paoletti M, Megna AS, Glielmo A, De Angelis M, Costa A, Meucci C, Calvaruso V, Sorrentini I, Palma G, Bruschi F, Nascetti G (2013) Anisakiasis and Gastroallergic Reactions Associated with Anisakis pegreffii Infection, Italy. Emerging Infectious Diseases 19(3):496-499 doi:10.3201/eid1903.121017 |
| Mattiucci S, Paoletti M, Borrini F, Palumbo M, Palmieri RM, Gomes V, Casati A, Nascetti G (2011) First molecular identification of the zoonotic parasite Anisakis pegreffii (Nematoda: Anisakidae) in a paraffin-embedded granuloma taken from a case of human intestinal anisakiasis in Italy. Bmc Infectious Diseases 11 doi:10.1186/1471-2334-11-82 |
| Mattiucci S, Paoletti M, Colantoni A, Carbone A, Gaeta R, Proietti A, Frattaroli S, Fazii P, Bruschi F, Nascetti G (2017) Invasive anisakiasis by the parasite Anisakis pegreffii (Nematoda: Anisakidae): diagnosis by real-time PCR hydrolysis probe system and immunoblotting assay. Bmc Infectious Diseases 17 doi:10.1186/s12879-017-2633-0 |
| Mejia-Renteria HD, Viana-Tejedor A, Sanchez-Enrique C, Nombela L, Paulo Herrera J, Ruiz-Mateos B, Javier Nunez-Gil I, Vivas D, Fernandez-Ortiz A, Macaya C (2014) Kounis syndrome after ingestion of undercooked fish: New role of intracoronary imaging techniques. International Journal of Cardiology 177(2):E58-E60 doi:10.1016/j.ijcard.2014.09.161 |
| Menendez P, Pardo R, Delgado M, Leon C (2015) Mesenteric tumor due to chronic anisakiasis. Revista Espanola De Enfermedades Digestivas 107(9):570-572 |
| Menghi CI, Comunale E, Gatta CL (2011) Anisakiosis: primer diagnóstico en Buenos Aires, Argentina. Revista de la Sociedad Venezolana de Microbiología 31(1):71-73 |
| Mercado P R, Torres H P, Gil L LC, Goldin G L (2006) Anisakiasis in a patient with a small hiatal hernia. Report of one case. Revista Medica De Chile 134(12):1562-1564 |
| Mercado R, Torres P, Munoz V, Apt W (2001) Human infection by Pseudoterranova decipiens (Nematoda, Anisakidae) in Chile: Report of seven cases. Memorias Do Instituto Oswaldo Cruz 96(5):653-655 doi:10.1590/s0074-02762001000500010 |
| Meseguer J, Navarro V, Sanchez-Guerrero I, Bartolome B, Alvarez JMN (2007) Anisakis simplex allergy and nephrotic syndrome. Allergologia Et Immunopathologia 35(5):216-220 doi:10.1157/13110318 |
| Minamoto T, Sawaguchi K, Ogino T, Mai M (1991) Anisakiasis of the colon - report of 2 cases with emphasis on the diagnostic and therapeutic value of colonoscopy. Endoscopy 23(1):50-52 doi:10.1055/s-2007-1010609 |
| Mineta S, Shimanuki K, Sugiura A, Tsuchiya Y, Kaneko M, Sugiyama Y, Akimaru K, Tajiri T (2006) Chronic anisakiasis of the ascending colon associated with carcinoma. Journal of Nippon Medical School = Nippon Ika Daigaku zasshi 73(3):169-74 |
| Mirabal SC, Theprungsirikul P, Zullow S, Sterling S, Kim J (2020) Opening a can of worms: Anisakiasis following raw fish consumption. Journal of General Internal Medicine 35(SUPPL 1):S531-S532 |
| MITANI M, SUGIURA M, KONDOH K (1994) A case of intussusception caused by anisakiasis of the sigmoid colon. The journal of the Japanese Practical Surgeon Society 55(11):2841-2844 |
| Mitsuboshi A, Yamaguchi H, Ito Y, Mizuno T, Tokoro M, Kasai M (2017) Extra-gastrointestinal anisakidosis caused by Pseudoterranova azarasi manifesting as strangulated inguinal hernia. Parasitology International 66(6):810-812 doi:10.1016/j.parint.2017.09.008 |
| Miura T, Iwaya A, Shimizu T, Tsuchiya J, Nakamura J, Yamada S, Miura T, Yanagi M, Usuda H, Emura I, Takahashi T (2010) Intestinal anisakiasis can cause intussusception in adults: An extremely rare condition. World Journal of Gastroenterology 16(14):1804-1807 doi:10.3748/wjg.v16.i14.1804 |
| Mizumura N, Okumura S, Tsuchihashi H, Ogawa M, Kawasaki M (2018) A Second Attack of Anisakis: Intestinal Anisakiasis Following Gastric Anisakiasis. Acg Case Reports Journal 5 doi:10.14309/crj.2018.65 |
| Mladineo I, Popovic M, Drmic-Hofman I, Poljak V (2016) A case report of Anisakis pegreffii (Nematoda, Anisakidae) identified from archival paraffin sections of a Croatian patient. Bmc Infectious Diseases 16 doi:10.1186/s12879-016-1401-x |
| Montalto M, Miele L, Marcheggiano A, Santoro L, Curigliano V, Vastola M, Gasbarrini G (2005) Anisakis infestation: a case of acute abdomen mimicking Crohn's disease and eosinophilic gastroenteritis. Digestive and Liver Disease 37(1):62-64 doi:10.1016/j.dld.2004.05.014 |
| Morikawa D, Hiraoka E (2014) Sushi worm: a case of anisakiasis. Journal of Gastroenterology and Hepatology 29:115-115 |
| Morishima R (2021) Reply to "Acute scrotum in a 8-year-old Italian child caused by extraintestinal anisakiasis in a seaside area". Allergy 76(5):1603-1603 doi:10.1111/all.14572 |
| Moschella CM, Mattiucci S, Mingazzini P, De Angelis G, Assenza M, Lombardo F, Monaco S, Paggi L, Modini C (2004) Intestinal anisakiasis in Italy: case report. Journal of Helminthology 78(3):271-273 doi:10.1079/joh2004237 |
| Moschella CM, Mattiucci S, Mingazzini P, Mongardini M, Chein A, Miccolis D, Modini C (2005) Intestinal anisakiasis in Italy: a case treated by emergency surgery. Giornale Di Chirurgia 26(5):201-205 |
| Mudry J, Lefebvre P, Deicas E, Vernes A, Poirriez J, Debat M, Marti R, Binot P, Cortot A (1986) Human anisakiasis - 5 new cases in the north of france. Gastroenterologie Clinique Et Biologique 10(1):83-87 |
| Muguruma N, Okamura S, Okahisa T, Shibata H, Ito S, Terauchi A (1999) Anisakis larva involving the esophageal mucosa. Gastrointestinal Endoscopy 49(5):653-654 doi:10.1016/s0016-5107(99)70401-3 |
| Mumoli N, Merlo A (2013) Colonic anisakiasis. Canadian Medical Association Journal 185(13):E652-E652 doi:10.1503/cmaj.120909 |
| Munoz S, Nuevo JA, Ruiz M, Gonzalez-Castillo J, Cubo P, Buron MR (2002) Acute abdominal pain and ascites in a young woman. Enfermedades Infecciosas Y Microbiologia Clinica 20(7):365-367 doi:10.1016/s0213-005x(02)72816-4 |
| Murakami D, Harada H, Amano Y (2020) Foreign body in the ileum after eating shabu-shabu. Postgraduate Medical Journal 96(1135):301-301 doi:10.1136/postgradmedj-2019-137200 |
| Murakami T, Takahashi A, Kimura R, Baba T, Satoi A, Makino T, et al. (2018) A case of ileal anisakiasis in which the anisakis larva was removed by colonoscopy [in Japanese]. . Prog Dig Endosc 92(1):128–129. also reported in Okamoto T, Fukuda K (2021) Anisakiasis of the Terminal Ileum Removed by Colonoscopy. Case Reports in Gastroenterology 15(1):47-52 doi:10.1159/000509954 |
| Muraoka A, Suehiro I, Fujii M, Nagata K, Kusunoki H, Kumon Y, Shirasaka D, Hosooka T, Murakami K (1996) Acute gastric anisakiasis - 28 cases during the last 10 years. Digestive Diseases and Sciences 41(12):2362-2365 doi:10.1007/bf02100128 |
| Murata Y, Ando K, Usui M, Sugiyama H, Hayashi A, Tanemura A, Kato H, Kuriyama N, Kishiwada M, Mizuno S, Sakurai H, Isaji S (2018) A case of hepatic anisakiasis caused by Pseudoterranova decipiens mimicking metastatic liver cancer. Bmc Infectious Diseases 18 doi:10.1186/s12879-018-3540-8 |
| Muwanwella N, Shimamura Y, Akram H, Kortan P, Marcon N (2016) Endoscopic diagnosis of gastric anisakiasis and extraction of larvae. Gastrointestinal Endoscopy 84(3):528-528 doi:10.1016/j.gie.2016.03.1504 |
| Muwanwella N, Shimamura Y, Marcon N (2016) A Rare Cause of Acute Abdomen. Clinical Gastroenterology and Hepatology 14(7):XXXV-XXXVI doi:10.1016/j.cgh.2016.02.028 |
| Na HK, Seo M, Chai JY, Lee EK, Jeon SM (2013) A case of anisakidosis caused by Pseudoterranova decipiens larva. Korean Journal of Parasitology 51(1):115-117 doi:10.3347/kjp.2013.51.1.115 |
| Najjari M, Sadjjadi SM, Khodadadi H, Farzaneh MR, Mattiucci S (2022) Anisakis spp, DNA detection in paraffin-embedded tissue biopsies recovered from patients with gastritis using real-time PCR in Bushehr, Persian Gulf, Iran. Molecular and Biochemical Parasitology 251 doi:10.1016/j.molbiopara.2022.111492 |
| Nakagawa Y, Nagai T, Okawara H, Nakashima H, Tasaki T, Soma W, Hisamatu A, Anan J, Murakami K, Fujioka T (2009) Comparison of magnified endoscopic images of Ancylostoma duodenale (hookworm) and Anisakis simplex. Endoscopy 41:E189-E189 doi:10.1055/s-0029-1214766 |
| Nakaji K (2009) Enteric Anisakiasis Which Improved with Conservative Treatment. Internal Medicine 48(7):573-573 doi:10.2169/internalmedicine.48.1905 |
| Nakaji K, Kumamoto M, Wada Y, Nakae Y (2019) Asymptomatic Gastric and Colonic Anisakiasis Detected Simultaneously. Internal Medicine 58(15):2263-2264 doi:10.2169/internalmedicine.2657-19 |
| Nakajo M, Setoguchi Y, Onohara S, Nakajo M (2011) Computed tomographic features of two cases of acute gastric anisakiasis. Abdominal Imaging 36(5):509-513 doi:10.1007/s00261-010-9646-0 |
| Nakamura H, Takanashi K, Morita R, Sakurada A, Hirata Y, Komatsu Y, Katsuki S (2022) A case of anisakiasis in the sigmoid colon. Clinical Case Reports 10(2):e05445-e05445 doi:10.1002/ccr3.5445 |
| Nakanishi Y, Yazumi S, Ikeuchi D, Matsumoto A, Yamamura H, Yoshizaki S, Mise S, Fukuyama N, Shimamoto C, Katsu K (2005) Vanishing gastric tumor caused by anisakiasis. Gastrointestinal Endoscopy 61(1):102-102 doi:10.1016/s0016-5107(04)02201-1 |
| Navarro Suarez EC, Algaba Montes M, Oviedo Garcia AA, Salgado Nevado V (2014) Anisakiasis: a disease that is underdiagnosed because clinical suspicion is low. Emergencias 26(2):159-160 |
| Nishikawa Y, Tsubokura M, Kato S, Saito Y (2014) Possible Anisakiasis Associated with Fishery Resumption. Disaster Medicine and Public Health Preparedness 8(2):117-118 doi:10.1017/dmp.2014.14 |
| Nogami Y, Fujii-Nishimura Y, Banno K, Suzuki A, Susumu N, Hibi T, Murakami K, Yamada T, Sugiyama H, Morishima Y, Aoki D (2016) Anisakiasis mimics cancer recurrence: two cases of extragastrointestinal anisakiasis suspected to be recurrence of gynecological cancer on PET-CT and molecular biological investigation. Bmc Medical Imaging 16 doi:10.1186/s12880-016-0134-z |
| Noh JH, Kim B-J, Kim SM, Ock M-S, Park MI, Goo JY (2003) A case of acute gastric anisakiasis provoking severe clinical problems by multiple infection. The Korean journal of parasitology 41(2):97-100 |
| Ogata M, Tamura S, Matsunoya M (2015) Sonographic Diagnosis of Intestinal Anisakiasis Presenting as Small Bowel Obstruction. Journal of Clinical Ultrasound 43(5):283-287 doi:10.1002/jcu.22194 |
| Okai T, Mouri I, Yamaguchi Y, Ohta H, Motoo Y, Sawabu N (1993) Acute gastric anisakiasis - observations with endoscopic ultrasonography. Gastrointestinal Endoscopy 39(3):450-452 doi:10.1016/s0016-5107(93)70129-7 |
| Okamoto T, Fukuda K (2021) Anisakiasis of the Terminal Ileum Removed by Colonoscopy. Case Reports in Gastroenterology 15(1):47-52 doi:10.1159/000509949 |
| Okano K, Oshima M, Suzuki Y (2010) Acute Abdomen With Epigastric Pain and Vomiting in an Adult Healthy Patient Acute Abdomen Caused by Anisakiasis of the Small Intestine. Gastroenterology 139(5):1465-1797 doi:10.1053/j.gastro.2009.11.060 |
| Oomori S, Kikuiri S, Ono S (2008) Gastric anisakiasis associated with bleeding gastric ulcer. Indian Journal of Gastroenterology: Official Journal of the Indian Society of Gastroenterology 27(3):129-129 |
| Ozcan HN, Avcu S, Pauwels W, Mortele KJ, De Backer AJ (2012) Acute intestinal anisakiasis: CT findings. Acta Gastro-Enterologica Belgica 75(3):364-365 |
| Pampiglione S, Rivasi F, Criscuolo M, De Benedittis A, Gentile A, Russo S, Testini M, Villani M (2002) Human anisakiasis in Italy: A report of eleven new cases. Pathology Research and Practice 198(6):429-434 doi:10.1078/0344-0338-00277 |
| Park M, Sun-Ja P, Chang HK, Seung OK, 최바울, 허진욱, 임현정, 이지영, 김동완, 구자영 (2003) A case of gastric submucosal tumor suspected to be caused by Anisakis. Clinical Endoscopy 27(1):26-30 |
| Park SW, Joo YE, Jung PJ, Ho PM, Lee NH, Jhu IK, Park GS, Park CH, Lee WS, Kim HS, Choi SK, Rew JS, Kim SJ (2006) Three cases of gastric anisakiasis mimicking submucosal tumor. Clinical Endoscopy 32(6):381-386 |
| Patino JA, Olivera MJ (2019) Gastro-allergic anisakiasis: The first case reported in Colombia and a literature review. Biomedica : revista del Instituto Nacional de Salud 39(2):241-246 doi:10.7705/biomedica.v39i2.3936 |
| Pellegrini M, Occhini R, Tordini G, Vindigni C, Russo S, Marzocca G (2005) Acute abdomen due to small bowel anisakiasis. Digestive and Liver Disease 37(1):65-67 doi:10.1016/j.dld.2004.06.018 |
| Petithory JC, Paugam B, Buyetrousset P, Paugam A (1990) Anisakis simplex, a cofactor of gastriccancer. Lancet 336(8721):1002-1002 doi:10.1016/0140-6736(90)92459-u |
| Pezzilli R, Casadei R, Santini D (2007) Autoimmune pancreatitis associated with anisakis infection. Digestive and Liver Disease 39(3):273-273 doi:10.1016/j.dld.2006.10.006 |
| Picard D, Herbinet B, Alexandre JH (1979) Acute abdominal syndromes and anisakiasis - new case. Annales De Chirurgie 33(1):40-44 |
| Pinel C, Beaudevin M, Chermette R, Grillot R, AmbroiseThomas P (1996) Gastric anisakidosis due to Pseudoterranova decipiens larva. Lancet 347(9018):1829-1829 doi:10.1016/s0140-6736(96)91648-7 |
| Pinkus GS, Coolidge C, Little MD (1975) Intestinal anisakiasis - 1st case report from North-America. American Journal of Medicine 59(1):114-120 doi:10.1016/0002-9343(75)90328-9; Richman RH, Lewicki AM (1973) Right Ileocolitis Secondary To Anisakiasis. American Journal of Roentgenology 119(2):329-331 doi:10.2214/ajr.119.2.329 |
| Piscaglia AC, Ventura MT, Landolfo G, Giordano M, Russo S, Landi R, Zulian V, Forte F, Stefanelli ML (2014) Chronic anisakidosis presenting with intestinal intussusception. European Review for Medical and Pharmacological Sciences 18(24):3916-3920 |
| Pontone S, Leonetti G, Guaitoli E, Mocini R, Manfredelli S, Catania A, Pontone P, Sorrenti S (2012) Should the host reaction to anisakiasis influence the treatment? Different clinical presentations in two cases. Revista Espanola De Enfermedades Digestivas 104(11):607-610 doi:10.4321/s1130-01082012001100010 |
| **Prasad S, D'Assumpcao C, McPheeters R, Stull W, Heidari A (2022) A case of Pseudoterranova, having ceviche with special flavor. Journal of Investigative Medicine 70(1):203-203 doi:10.1136/jim-2022-WRMC.182** |
| Qin Y, Zhao Y, Ren Y, Zheng L, Dai X, Li Y, Mao W, Cui Y (2013) Anisakiasis in China: The first clinical case report. Foodborne Pathogens and Disease 10(5):472-474 doi:10.1089/fpd.2012.1325 |
| Ramanan P, Blumberg AK, Mathison B, Pritt BS (2013) Parametrial Anisakidosis. Journal of Clinical Microbiology 51(10):3430-3434 doi:10.1128/jcm.01398-13 |
| Ramos L, Alonso C, Guilarte M, Vilaseca J, Santos J, Malagelada JR (2005) Anisakis simplex-induced small bowel obstruction after fish ingestion: Preliminary evidence for response to parenteral corticosteroids. Clinical Gastroenterology and Hepatology 3(7):667-671 doi:10.1016/s1542-3565(05)00363-0 |
| Rea R, Di Matteo F, Scarpa F, Pandolfi M, Martino M, Dicuonzo G, Gabbrielli A (2008) Identification and removal of an Anisakis in acute gastric anisakiasis. Digestive and Liver Disease 40:S73-S73 doi:10.1016/s1590-8658(08)60187-5 |
| Repiso Ortega A, Alcantara Torres M, Gonzalez de Frutos C, de Artaza Varasa T, Rodriguez Merlo R, Valle Munoz J, Martinez Potenciano JL (2003) Gastrointestinal anisakiasis. Study of a series of 25 patients. Gastroenterologia Y Hepatologia 26(6):341-6 doi:10.1157/13048887 |
| Rezapour M, Agarwal N (2017) You Are What You Eat: A Case of Nematode-Induced Eosinophilic Esophagitis. Acg Case Reports Journal 4 doi:10.14309/crj.2017.13 |
| Riu Pons F, Gimeno Beltran J, Albero Gonzalez R, Alvarez Gonzalez MA, Dedeu Cusco JM, Barranco Priego L, Seoane Urgorri A (2015) An unusual presentation of anisakiasis in the colon (with video). Gastrointestinal Endoscopy 81(4):1050-1051 doi:10.1016/j.gie.2014.10.017 |
| Roca-Gerones X, Magdalena Alcover M, Godinez-Gonzalez C, Gonzalez-Moreno O, Masachs M, Fisa R, Montoliu I (2020) First Molecular Diagnosis of Clinical Cases of Gastric Anisakiosis in Spain. Genes 11(4) doi:10.3390/genes11040452 |
| Romeo Ramirez JA, Martinez-Conde Lopez AE, Olivares Galdeano U, Sancha Perez A, Lopez de Torre Ramirez de la Piscina J, Barros Ingerto J, Echavarri Inigo J (1997) Gastric anisakiasis diagnosed by endoscopy. Gastroenterologia Y Hepatologia 20(6):306-8 |
| Rosales J, Mascaro C, Fernandez C, Luque F, Moreno MS, Parras L, Cosano A, Munoz JR (1999) Acute intestinal anisakiasis in Spain: a fourth-stage Anisakis simplex larva. Memorias Do Instituto Oswaldo Cruz 94(6):823-826 doi:10.1590/s0074-02761999000600020 |
| Ruan W, Berger DM, Kellermayer R (2020) Asymptomatic Anisakiasis in a Patient With Crohn Disease. Journal of Pediatric Gastroenterology and Nutrition 71(2):E72-E72 doi:10.1097/mpg.0000000000002696 |
| Ruiz de la Hermosa A, Ortiz Johansson C, Luisa de Fuenmayor M, Casado Farinas I, Seoane Gonzalez JB (2017) Colonic intussusception caused by anisakiasis. A rare cause of obstruction. Gastroenterologia Y Hepatologia 40(10):680-682 doi:10.1016/j.gastrohep.2016.09.002 |
| Rushovich AM, Randall EL, Caprini JA, Westenfelder GO (1983) Omental anisakiasis - a rare mimic of acute appendicitis. American Journal of Clinical Pathology 80(4):517-520 doi:10.1093/ajcp/80.4.517 |
| Saito H, Nawata Y, Hirasawa D (2021) Posterior Mediastinitis Caused by Esophageal Anisakiasis. Clinical Gastroenterology and Hepatology 19(7):XXX-XXX doi:10.1016/j.cgh.2020.04.063 |
| Sakanari JA, Loinaz HM, Deardorff TL, Raybourne RB, McKerrow JH, Frierson JG (1988) Intestinal anisakiasis - a case diagnosed by morphologic and immunological methods. American Journal of Clinical Pathology 90(1):107-113 doi:10.1093/ajcp/90.1.107 |
| Sanchez Justicia C, Granero Peiro L, Arabe Paredes JA (2017) Anisakiasis and intestinal endometriosis: under-recognized conditions in the differential diagnosis of acute abdomen. Revista espanola de enfermedades digestivas : organo oficial de la Sociedad Espanola de Patologia Digestiva 109(1):81-82 doi:10.17235/reed.2016.4393/2016 |
| Sapunar J, Doerr E, Letonja T (1976) Human anisakiasis in Chile (author's transl). Boletin chileno de parasitologia 31(3-4):79-83 |
| Sasaki T, Fukumori D, Matsumoto H, Ohmori H, Yamamoto F (2003) Small bowel obstruction caused by anisakiasis of the small intestine: Report of a case. Surgery Today 33(2):123-125 doi:10.1007/s005950300027 |
| Sashiyama H, Fu KI, Hoshino T, Tsujinaka Y (2010) Gastrointestinal: Gastric anisakiasis presenting as a submucosal tumour diagnosed by endoscopic submucosal dissection. Journal of Gastroenterology and Hepatology 25(11):1806-1806 doi:10.1111/j.1440-1746.2010.06519.x |
| Sato T, Ogata H, Hosoda Y, Esaki T, Inoue N, Ishii H (2001) A case of ileal anisakiasis removed by colonoscopy. Progress of Digestive Endoscopy 58(2):112-113 doi:10.11641/pde.58.2_112 |
| saw in Suankratay C, Wilde H, Berger S (2001) Thailand: Country survey of infectious diseases. Journal of Travel Medicine 8(4):192-203 |
| Schuster R, Petrini JL, Choi R (2003) Anisakiasis of the colon presenting as bowel obstruction. American Surgeon 69(4):350-352 |
| Seike T, Kobayashi M, Suda T, Oishi N (2020) A Case of Anaphylaxis with Mild Abdominal Pain Due to Gastroallergic Anisakiasis. American Journal of Medicine 133(10):E601-E602 doi:10.1016/j.amjmed.2020.02.054 |
| Sekimoto M, Nagano H, Fujiwara Y, Watanabe T, Katsu K, Doki Y, Mori M (2011) Two Cases of Gastric Anisakiasis for which Oral Administration of a Medicine Containing Wood Creosote (Seirogan (R)) was Effective. Hepato-Gastroenterology 58(109):1252-1254 |
| Shamsi S, Butcher AR (2011) First report of human anisakidosis in Australia. Medical Journal of Australia 194(4):199-200 doi:10.5694/j.1326-5377.2011.tb03772.x |
| Shigeno A, Funakoshi H (2022) Images in emergency medicine, Gastric anisakiasis. Annals of Emergency Medicine 79(2):E5-E6 |
| Shikino K, Ikusaka M (2019) Anaphylaxis Induced by Anisakis. Internal Medicine 58(14):2121-2121 doi:10.2169/internalmedicine.2428-18 |
| Shimamura Y, Ishii N, Ego M, Nakano K, Ikeya T, Nakamura K, Takagi K, Fukuda K, Fujita Y (2016) Multiple Acute Infection by Anisakis: A Case Series. Internal Medicine 55(8):907-910 doi:10.2169/internalmedicine.55.5628 |
| Shimamura Y, Muwanwella N, Chandran S, Kandel G, Marcon N (2016) Common Symptoms from an Uncommon Infection: Gastrointestinal Anisakiasis. Canadian journal of gastroenterology & hepatology 2016:5176502-5176502 |
| SHIMIZU T, YAMAZAKI K, BANDO T, TAJIKA S, TSUKADA K (2012) Anisakiasis of the ascending colon presenting as intussuscept. Japanese Journal of Clinical Surgery 73(5):1149-1153 doi:10.3919/jjsa.73.1149 |
| Shirahama M, Koga T, Uchida S, Miyamoto Y, Ohta Y, Ishibashi H (1990) Colonic anisakiasis simulating carcinoma of the colon. American Journal of Roentgenology 155(4):895-895 doi:10.2214/ajr.155.4.2119131 |
| Shudo R, Yazaki Y, Yamada H, Sugawara K, Takahashi K (2002) Infection with a black Pseudoterranova decipiens. Gastrointestinal Endoscopy 56(1):110-110 doi:10.1067/mge.2002.123911 |
| Shweiki E, Rittenhouse DW, Ochoa JE, Punja VP, Zubair MH, Baliff JP (2014) Acute Small-Bowel Obstruction From Intestinal Anisakiasis After the Ingestion of Raw Clams; Documenting a New Method of Marine-to-Human Parasitic Transmission. Open Forum Infectious Diseases 1(2) doi:10.1093/ofid/ofu087 |
| Skirnisson K (2006) Pseudoterranova decipiens (Nematoda, Anisakidae) larvae reported from humans in Iceland after consumption of insufficiently cooked fish. Laeknabladid 92(1):21-5 |
| Skirnisson K (2022) Human Pseudoterranova and Anisakis cases in Iceland 2004-2020. Laeknabladid 108(2):79-83 doi:10.17992/lbl.2022.02.676 |
| Sohn WM, Seol SY (1994) A human case of gastric anisakiasis by Pseudoterranova decipiens larva. The Korean journal of parasitology 32(1):53-6 |
| Sohn, W.-M.; Na, B.-K.; Kim, T.H.; Park, T.-J. Anisakiasis: Report of 15 Gastric Cases Caused by Anisakis Type I Larvae and a Brief Review of Korean Anisakiasis Cases. Korean Journal of Parasitology 2015, 53, 465-470, doi:10.3347/kjp.2015.53.4.465. |
| Soon-Young S, Hee KB, 김위경, 조온구, 김용수, 정우경, 김민영 (2011) CT Findings of Small Bowel Anisakiasis: Analysis of Four Cases. Journal of the Korean Society of Radiology (JKSR) 64(2):167-171 |
| Spehn J, Schonbeck S, Koperski K, Butzow GH (1988) Atypical anisakiasis in a patient with AIDS. Deutsche Medizinische Wochenschrift 113(24):983-984 doi:10.1055/s-2008-1067754 |
| Sugano S, Suzuki T, Kagesawa M, Kawafune T, Ohshima Y (1993) Noncardiac chest pain due to acute gastric anisakiasis. Digestive Diseases and Sciences 38(7):1354-1356 doi:10.1007/bf01296091 |
| SUGIYAMA S, TANIGAWA T (2000) A case of anisakiasis of the colon causing intussusception. Nihon Rinsho Geka Gakkai Zasshi (Journal of Japan Surgical Association) 61(3):714-71 |
| Suh YA, Jang HJ, Eun CS, Jang WY, Lee JJ, Kae SH, Lee J (2004) A case of a submucosal tumor in the ascending colon probably caused by Anisakis. Clinical Endoscopy 28(4):202-207 |
| Suzuki S, Bandoh N, Goto T, Uemura A, Sasaki M, Harabuchi Y (2021) Severe laryngeal edema caused by Pseudoterranova species A case report. Medicine 100(4) doi:10.1097/md.0000000000024456 |
| Tachizawa N, Tajima H, Adachi M, Isono A, Mikami M, Sagawa T, Tanaka A, Sakamoto T, Takikawa H, Yamamoto A, Takeshita K (2012) Six cases with ileus caused by small bowel anisakiasis, diagnosed by abdominal CT scanning and treated without surgical operation. Journal of Gastroenterology and Hepatology 27:397-397 |
| Takabe K, Ohki S, Kunihiro O, Sakashita T, Endo I, Ichikawa Y, Sekido H, Amano T, Nakatani Y, Suzuki K, Shimada H (1998) Anisakidosis: A cause of intestinal obstruction from eating sushi. American Journal of Gastroenterology 93(7):1172-1173 |
| Takahara H, Takahara S (2013) A case of ileal anisakiasis in which the anisakis larva was removed with double-balloon endoscopy [in Japanese]. Gastroenterol Endosc 55:22–27 |
| Takamizawa Y, Kobayashi Y (2015) Adhesive Intestinal Obstruction Caused by Extragastrointestinal Anisakiasis. American Journal of Tropical Medicine and Hygiene 92(4):675-676 doi:10.4269/ajtmh.14-0673 |
| Takano K, Okuni T, Murayama K, Himi T (2016) A Case Study of Anisakiasis in the Palatine Tonsils. Advances in oto-rhino-laryngology 77:125-7 doi:10.1159/000441903 |
| Takano Y, Gomi K, Endo T, Suzuki R, Hayashi M, Nakanishi T, Tateno A, Yamamura E, Asonuma K, Ino S, Kuroki Y, Nagahama M, Inoue K, Takahashi H (2013) Small intestinal obstruction caused by anisakiasis. Case Reports in Infectious Diseases 2013 doi:10.1155/2013/401937 |
| Takasaki T, Yamada T, Kinoshita J, Motomura Y (2020) Asymptomatic Colonic Anisakiasis: Is It So Rare? Case Reports in Gastroenterology 14(3):593-597 doi:10.1159/000508822 |
| Takei H, Powell SZ (2007) Intestinal anisakidosis (anisakiosis). Annals of Diagnostic Pathology 11(5):350-352 doi:10.1016/j.anndiagpath.2006.03.018 |
| Takeuchi K, Hanai H, Iida T, Suzuki S, Isobe S (2000) A bleeding gastric ulcer on a vanishing tumor caused by anisakiasis. Gastrointestinal Endoscopy 52(4):549-551 doi:10.1067/mge.2000.108527 |
| Tamai Y, Kobayashi K (2015) Asymptomatic Colonic Anisakiasis. Internal Medicine 54(6):675-675 doi:10.2169/internalmedicine.54.3649 |
| Tanaka A, Kanmura S, Sakiyama T, Ido A (2021) Huge gastric lesion disappears in a day. Digestive and Liver Disease 53(10):1359-1359 doi:10.1016/j.dld.2020.08.006 |
| Taniguchi G, Nagahara A, Matsumoto K, Ritsuno H, Igusa Y, Sasaki H, Mori H, Beppu K, Shibuya T, Sakamoto N, Osada T, Kawabe M, Terai T, Ogihara T, Watanabe S (2011) Asymptomatic anisakiasis of the colon incidentally found by colonoscopy. Clinical Journal of Gastroenterology 4(6):371-373 doi:10.1007/s12328-011-0247-4 |
| Tantaleán V M, Huiza F A (1993) Nematode larvae with medical importance found in sea fish from the peruvian shore, with two records of human infections. Rev peru med trop:61-5 |
| Tokuyama H, Nakayama Y, Tsuno M, Obayashi M, Nanjoh T, Kin H, Yanagawa K, Kawaguchi M, Nishimori T, Sakazaki S, Han N, Kuroki T (2000) A case of anisakiasis of the rectum treated under colonoscopy. Nihon Shokakibyo Gakkai zasshi = The Japanese journal of gastro-enterology 97(5):616-8 |
| Torre I, Gutierrez-Macias A, Elorriaga K, Martin E, Zubero Z, Santamaria JM (2002) Intestinal occlusion in a previously healthy woman. Enfermedades Infecciosas Y Microbiologia Clinica 20(10):531-532 doi:10.1016/s0213-005x(02)72857-7 |
| Torres M, Canales M, Concha M, Cofre X, Tellez P. Un caso de anisakiosis en un adulto. Parasitol. día. 2000;24(3-4):1-4. https://doi.org/dnw77b |
| Torres P, Jercic MI, Weitz JC, Dobrew EK, Mercado RA (2007) Human pseudoterranovosis, an emerging infection in Chile. Journal of Parasitology 93(2):440-443 doi:10.1645/ge-946r.1 |
| Toyoda H, Tanaka K (2016) Intestinal Anisakiasis Treated Successfully with Prednisolone and Olopatadine Hydrochloride. Case Reports in Gastroenterology 10(1):30-5 doi:10.1159/000442971 |
| Tsuji K, Ikeda H, Mitani H, Saito M, Ando T, Hirakawa E (2003) A case of small intestinal intussusception caused by anisakidosis. Japanese Journal of Gastroenterological Surgery 36(5):422-426 |
| Tsukamoto H, Koizumi W, Atari E, Okudaira M, Yoshizawa S, Maekawa K, Mieno H, Hamashima H, Ishikawa J, Takahashi T (1987) A case of gastric anisakiasis, causing gastric perforation. Nihon Shokakibyo Gakkai zasshi = The Japanese journal of gastro-enterology 84(1):104-7 |
| Tsukui M, Morimoto N, Kurata H, Sunada F. Asymptomatic anisakiasis of the colon incidentally diagnosed and treated during colonoscopy by retroflexion in the ascending colon. J Rural Med. 2016;11(2):73–5. |
| Tsynman D, Wang LS, Zhang L, Yiu P, Ng AJ (2015) Think Twice With That Tuna Tartare. American Journal of Gastroenterology 110:S201-S201 doi:10.14309/00000434-201510001-00466 |
| Ueda A, Tori A, Yoshimura T, Kakui K, Ishikawa Y (2017) A Case of Extra-Gastrointestinal Anisakidosis With Incidental Detection of a Live Larva During Laparoscopic Cystectomy. Journal of Minimally Invasive Gynecology 24(6) doi:10.1016/j.jmig.2016.12.003 |
| Uehara A, Okumura T (2017) Esophageal Anisakiasis Mimicking Gastroesophageal Reflux Disease. American Journal of Gastroenterology 112(4):532-532 doi:10.1038/ajg.2016.516 |
| Una-Gorospe M, Herrera-Mozo I, Luisa Canals M, Marti-Amengual G, Sanz-Gallen P (2018) Occupational disease due to Anisakis simplex in fish handlers. International Maritime Health 69(4):264-269 doi:10.5603/imh.2018.0042 |
| Urita Y, Nishino M, Koyama H, Kondo E, Naruki Y, Otsuka S (1997) Esophageal anisakiasis accompanied by reflux esophagitis. Internal Medicine 36(12):890-893 doi:10.2169/internalmedicine.36.890 |
| Vallejo Rodriguez MC, Valero Tena E, Charro Calvillo M, Mur Villacampa M (2006) Recurrent abdominal pain: gastric and ileal manifestations by Anisakis. Anales de medicina interna (Madrid, Spain : 1984) 23(11):556-7 |
| van Thiel P, Jansen J (1987) A parasitic infection in a dutch woman, probably caused by the consumption of imported trout. Acta Leidensia 56:57-63 |
| Vaughan S, Sadler M, Jayakumar S, Missaghi B, Chan W, Church DL (2015) An unusual case of abdominal pain. Canadian Journal of Infectious Diseases & Medical Microbiology 26(6):297-298 doi:10.1155/2015/578715 |
| Verhamme MAM, Ramboer CHR (1988) Anisakiasis caused by herring in vinegar - a little known medical problem. Gut 29(6):843-847 doi:10.1136/gut.29.6.843 |
| Verma R, Gogia JS, Khalili A (2005) Ceviche and abdominal pain: A rare case of Anisakis simplex in the United States. American Journal of Gastroenterology 100(9):S191-S191 doi:10.14309/00000434-200509001-00505 |
| Vila Costas JJ, Martinez-Penuela Virseda JM, Zozaya Urmeneta JM, Manrique Celada M, Aznarez Barrio R, Zozaya Alvarez E, Borda Celaya F (2003) Brunner gland hamartoma and anisakiasis: etiologic association? Anales de medicina interna (Madrid, Spain : 1984) 20(9):470-2 |
| Vinsard DG, Raimondo M (2018) Gastric anisakiasis Commentary. Gastrointestinal Endoscopy 88(4):767-767 |
| Vuong PN, Montbrun TDP, Lemarchand N, Ganansia R, Meyrignac P, Houissa-Vuong S (2000) Anisakiasis of the small intestine mimicking a metastatic tumor: a new French case. Medecine Et Maladies Infectieuses 30(8):528-532 doi:10.1016/s0399-077x(00)80017-7 |
| Wang E, Kaur N, Saad R (2008) Acute duodenal anisakiasis with associated hepatopancreatic complications. American Journal of Gastroenterology 103:S303-S303 doi:10.14309/00000434-200809001-00769 |
| Watanabe T, Ohta S, Iwamoto S, Tsuji Y, Morita S, Doi I, Ueda Y, Chiba T (2008) Small Bowel Anisakiasis with Self-limiting Clinical Course. Internal Medicine 47(24):2191-2192 doi:10.2169/internalmedicine.47.1675 |
| Watanabe T, Tokuda Y (2014) Small Bowel Obstruction Following the Consumption of "Sushi". Internal Medicine 53(22):2661-2662 doi:10.2169/internalmedicine.53.3583 |
| Watanabe T, Tokuda Y (2014) Small Bowel Obstruction Following the Consumption of "Sushi". Internal Medicine 53(22):2661-2662 doi:10.2169/internalmedicine.53.3583 |
| Watt I, McLean N, Girdwood R, Kissen L, Fyfe AB (1979) Eosinophilic gastroenteritis associated with a larval anisakine nematode. Lancet 2:893-894 |
| Weitzel T, Sugiyama H, Yamasaki H, Ramirez C, Rosas R, Mercado R (2015) Human infections with Pseudoterranova cattani nematodes, Chile. Emerging Infectious Diseases 21(10):1874 |
| Yamada T, Ohwada S (2020) Case of gastric anisakiasis with no symptoms. Clinical Case Reports 8(9):1833-1834 doi:10.1002/ccr3.2948 |
| Yasumizu T, Hirano M, Nakano H, Tomonari K, Ii K, Tsunetomi N, et al. Endoscopic treatment of terminal ileal anisakiasis: a case report [in Japanese]. Gastroenterol Endosc. 1998;40:818–23. |
| Yeum CH, Ma SK, Kim SW, Kim NH, Kim J, Choi KC (2002) Incidental detection of an Anisakis larva in continuous ambulatory peritoneal dialysis effluent. Nephrology Dialysis Transplantation 17(8):1522-1523 doi:10.1093/ndt/17.8.1522 |
| Yokogawa M, Yoshimura H (1965) Anisakis-like larvae causing eosinophilic granulomata in stomach of man. American Journal of Tropical Medicine and Hygiene 14(5):770-773 doi:10.4269/ajtmh.1965.14.770 |
| Yokogawa M, Yoshimura H (1967) Clinicopathologic studies on larval anisakiasis in Japan. American Journal of Tropical Medicine and Hygiene 16(6):723-+ doi:10.4269/ajtmh.1967.16.723 |
| Yoo HJ, Kim SH, Lee JM, Kim MA, Han JK, Choi BI (2008) The association of anisakiasis in the ascending colon with sigmoid colon cancer: CT colonography findings. Korean Journal of Radiology 9:S56-S60 doi:10.3348/kjr.2008.9.s.s56 |
| Yoon WJ, Lee SM, Lee SH, Yoon YB (2004) Gastric anisakiasis. Gastrointestinal Endoscopy 59(3):400-400 doi:10.1016/s0016-5107(03)02591-4 |
| Yorimitsu N, Hiraoka A, Utsunomiya H, Imai Y, Tatsukawa H, Tazuya N, Yamago H, Shimizu Y, Hidaka S, Tanihira T, Hasebe A, Miyamoto Y, Ninomiya T, Abe M, Hiasa Y, Matsuura B, Onji M, Michitaka K (2013) Colonic Intussusception Caused by Anisakiasis: A Case Report and Review of the Literature. Internal Medicine 52(2):223-226 doi:10.2169/internalmedicine.52.8629 |
| Yoshikawa I, Murata I, Kanagawa K, Otsuki M (1996) A case of asymptomatic colonic anisakiasis revealed by chance and treated by colonoscopy. Nihon Shokakibyo Gakkai zasshi = The Japanese journal of gastro-enterology 93(11):837-40 |
| Young JE, Oh HJ, Kim TH, Choi S-C, Na Y-H, et al. (2006) Anisakiasis of the colon: Report of two cases. Clinical Endoscopy 32(4):298-301 |
| Yu JR, Seo M, Kim YW, Oh MH, Sohn WM (2001) A human case of gastric infection by Pseudoterranova decipiens larva. The Korean journal of parasitology 39(2):193-6 |
| Zanelli M, Ragazzi M, Fiorino S, Foroni M, Cecinato P, Sanchez MdMJ, Ascani S, de Marco L (2017) An Italian case of intestinal anisakiasis with a presurgical diagnosis: Could this parasite represent an emerging disease? Pathology Research and Practice 213(5):558-564 doi:10.1016/j.prp.2017.01.027 |
| Zullo A, Balsamo G, Baldini D, De Francesco V, Manta R (2018) Asymptomatic Anisakis and erosive lesions in the colon. Annals of Gastroenterology:246-246 |
| Zvejnieks PA, Lichtenstein KA, Koneman EW (1998) Photo quiz II - Diagnosis: Luminal anisakidosis due to Pseudoterranova decipiens. Clinical Infectious Diseases 26(5):1085-+ |
| Suh S, Suh B, Yu H, Lim S, Kim J (2005) Gastrointestinal Anisakiasis. Annals of Surgical Treatment and Research 69(5):417-419 |
